# Pilot-Scale Continuous Flow Synthesis of Capsaicinoids and Their Formulation with Cyclodextrins

**DOI:** 10.1021/acsomega.5c10910

**Published:** 2026-01-09

**Authors:** Bettina Rávai, Dóra V. Ujj, Máté J. Orosz, Ecaterina Revenco, Szabolcs Béni, Ádám Tajti, Erika Bálint

**Affiliations:** † Department of Organic Chemistry and Technology, Faculty of Chemical Technology and Biotechnology, 61810 Budapest University of Technology and Economics, Műegyetem rkp. 3., H-1111 Budapest, Hungary; ‡ CycloLab Cyclodextrin Research and Development Ltd., Illatos út 7., H-1097 Budapest, Hungary; § Department of Analytical Chemistry, Faculty of Science, 54616 Eötvös Loránd University, Pázmány Péter Sétány 1/a., H-1117 Budapest, Hungary; ∥ Bálint Analitika Ltd., Kondorfa u. 6., H-1116 Budapest, Hungary

## Abstract

Capsaicinoids are the principal compounds responsible for the pungency of chili peppers and are widely used as food additives as well as in pharmaceutical and cosmetic formulations or potential agrochemicals. However, capsaicin and its derivatives have poor aqueous solubility, which limits their broader application. In this study, we report the first pilot-scale continuous flow synthesis of capsaicin, dihydrocapsaicin, and nonivamide, using sequential oxime formation, hydrogenation, and *N*-acylation steps. To increase the water solubility of these capsaicinoids, we systematically investigated their inclusion complex formation with various α- and β-cyclodextrin (CD) derivatives. Phase-solubility analyses and stability constant determinations were conducted to evaluate the complexation efficiency. Furthermore, 1D and 2D NMR spectroscopy confirmed 1:1 host–guest stoichiometry and revealed key intermolecular interactions between the CDs and the aliphatic moieties of the capsaicinoids. Overall, these results provide a scalable synthetic pathway and an efficient formulation strategy for capsaicinoid-based applications, which are particularly valuable in the pharmaceutical industry, food production, or agricultural processing, where aqueous solubilization and safety compliance are critical due to the potential irritant effects of capsaicinoid-containing powders or sprays.

## Graphical abstract

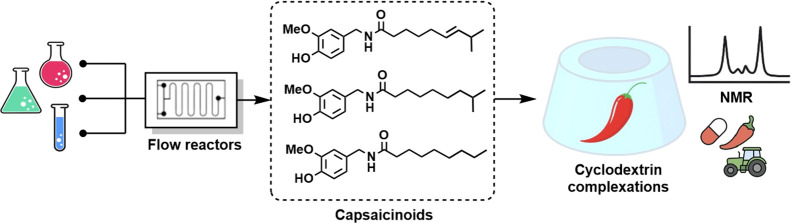

## 1 Introduction

Capsaicin, which is a unique alkaloid and the most essential capsaicinoid, is primarily found in the fruit of *Capsicum genus* and, therefore, responsible for its spicy flavor.^1–3^ Further capsaicinoids (as dihydrocapsaicin, nordihydrocapsaicin, homodihydrocapsaicin, homocapsaicin, and nonivamide), as the main derivatives of capsaicin, can still provide the strong pungency of chili pepper fruit.^4^

Capsaicin and its derivatives are widely utilized in the food industry not only for their spicy flavors but also for their functional properties. They exhibit antimicrobial and antioxidant activities, making them attractive candidates for natural food preservation. Their ability to inhibit the growth of certain spoilage-causing microorganisms and pathogens contributes to improved shelf life and food safety.^5^ In addition, capsaicinoids are also being investigated in functional foods and dietary supplements for their potential health benefits, such as metabolism stimulation and appetite control.^6^

Moreover, their medical use shows further possible beneficial effects in many diseases. While arthritis and musculoskeletal pain are FDA-labeled indications for capsaicin, it has also been reported that it is able to kill human prostate or colon cancer cells by forcing them to undergo apoptosis.^7,8^ Furthermore, it may exert potentially useful effects on glucose regulation, insulin homeostasis, and diabetes.^9^

In agriculture, capsaicinoids serve as natural deterrents against pests and herbivores due to their strong irritant effects.^10^ They are used in eco-friendly repellent formulations for crop protection and have also shown insecticidal, antibacterial (against *Salmonella typhimurium*, *Bacillus cereus,* or *Streptococcus mutans*), and antifungal activity (against *Colletotrichum capsici*, *Aspergillus niger,* and *Fusarium oxysporum*), which could offer a sustainable alternative to synthetic agrochemicals.^11,12^

Due to the aforementioned expanding range of applications, there has been a much higher demand for capsaicin and capsaicinoids in recent years. Generally, capsaicinoids are extracted directly from the fruit; however, there is a growing need for efficient organic synthesis approaches to increase their production scale.^13^ Several batch methods for their synthetic preparation are described in the literature; however, only one example of the pilot-scale production of capsaicin exists.^14,15^ As a previous result, our research group successfully developed the first flow synthesis of pure capsaicin and its derivatives, which provides a safer, faster, and more environmentally friendly process compared to the batch methods.^16^ The flow process consists of three reaction steps, such as oxime formation, reduction, and *N*-acylation, and its technological feasibility has also been tested in 3D printed flow reactors. However, the real potential for continuous synthesis of capsaicin and its bioactive analogues lies in pilot or industrial scale production, with process intensification and increased productivity.

Regarding the chemical properties of capsaicin, it is known that it is a lipophilic (fat-, alcohol-, and oil-soluble) chemical compound, so for its aqueous solubilization, it requires organic solvents, the use of surfactants, or inclusion complexation formulations with other molecules.^17–19^ In the pharmaceutical, food, and agriculture industries, these limitations restrict their use as functional ingredients or additives, and the irritating airborne particles of the powdered capsaicinoids can pose safety concerns for workers during formulation and application processes.

Cyclodextrin (CD)-based inclusion complexes provide a powerful strategy to overcome these challenges. CDs are a family of cyclic oligosaccharides, namely, glucose units linked by α-1,4-glycosidic bonds.^20^ They are produced through the enzymatic degradation of starch and are classified into three main types based on the number of glucose units: α-CD (6 units), β-CD (7 units), and γ-CD (8 units). These molecules have a unique toroidal structure with a hydrophobic inner cavity and a hydrophilic outer surface. This allows them to form inclusion complexes with a wide range of guest molecules, encapsulating hydrophobic compounds such as capsaicinoids within their cavity. As a result, it is possible to increase the aqueous solubility and thermal stability, enhance bioavailability, and simultaneously reduce the undesired tastes or odors of hydrophobic guest molecules.^21,22^

In the literature, there are some research studies that mention the importance of the inclusion complexation between capsaicin and CDs. For example, Chen et al. reported a study on the investigation of the formation of an inclusion complex between capsaicin and 2-hydroxypropyl-beta-cyclodextrin (HP-β-CD), in which pharmacokinetic studies were conducted in rats to compare bioavailability and subcutaneous absorption between free and complexed capsaicin.^23^ Two Chinese research groups further investigated the formation of the same inclusion complex, enhancing the solubility, thermal stability, and fluorescence properties of capsaicin.^24,25^ In another publication, it was proven that the combination of a local anesthetic with a capsaicin and HP-β-CD complex is a promising approach for effective anesthesia in inflamed conditions.^19^ Furthermore, it is known that the inclusion complex of capsaicin and α-CD can significantly improve the activity of antibiotic films against Gram-positive and Gram-negative strains.^26^ A further study reported a more unique application, the allosteric binding of capsaicin to a synthetic bis­(β-CD)-2,2′-bipyridine receptor, demonstrating a cooperative interaction between the two CD units.^27^

In this article, we report on the first pilot-scale flow chemical synthesis method for the preparation of capsaicin, dihydrocapsaicin, and nonivamide (synthetic capsaicin) through oxime formation, hydrogenation, and *N*-acylation steps. The efficiency of the scale-up was demonstrated by using continuous flow chemical reactors. A further aim was to investigate the inclusion complexation of capsaicinoids synthesized with different α- and β-CD derivatives. The comprehensive study of capsaicinoid-CD interactions is a novel contribution to the field of supramolecular chemistry, as to the best of our knowledge, no previous studies have utilized such a wide range of CDs and capsaicinoids together. In our study, both phase-solubility and complex stability calculations were performed, while the stoichiometry of the complex was determined by Job’s plot method using ^1^H NMR. The intermolecular interactions present in the host complexes were determined by 2D ROESY NMR measurements. By improving solubility and bioavailability and reducing the irritative effects of different capsaicinoids, our work opens up new potential opportunities for the pharmaceutical, food, agricultural, and cosmetic applications of capsaicinoids.

## 2 Experimental Section

### 2.1 Materials

Vanillin oxime (**3**), vanillylamine (**4**), capsaicin (**6a**), dihydrocapsaicin (**6b**) and nonivamide (**6c**) were synthesized according to the procedure reported in the Supporting Information. All the CDs were purchased from Cyclolab Ltd. (Budapest, Hungary) and were used without further purification, while deuterium oxide (D_2_O-deuteration degree min. 99.8%) and dimethyl sulfoxide-*d*_6_ (DMSO-*d*_6_-deuteration degree min. 99.8%) were obtained from Merck (Merck KGaA, Darmstadt, Germany).

### 2.2 Instruments

For the oxime formation and *N*-acylation steps, a Syrris Asia continuous syringe pump and a Syrris Asia heatable reactor were utilized. To apply flow to the system, “green” (500 and 250 μL) syringes were used in the syringe pump, unless otherwise stated. Thus, the total volume of the Syrris Asia flow system with the connection tubings was 5.6 mL, unless otherwise stated. The hydrogenation step was conducted in an H-Cube Pro continuous flow hydrogenating system developed by ThalesNano Inc. (with a total volume of 3.5 mL), supported by a Knauer Azura P 2.1S HPLC pump. For connections, standard 1″-28 fittings and 1/16″ poly­(tetrafluoroethylene) (PTFE) tubing (inner diameter = 0.5 mm) were used. Between the oxime formation step and the hydrogenation, a poly­(etheretherketone) (PEEK) T-mixer was installed. To avoid the evaporation of the solvents during the *N*-acylation, a 7 bar back pressure regulator (BPR) was connected after the Syrris Asia reactor.

High-performance liquid chromatography–mass spectrometry (HPLC-MS) measurements were performed with an Agilent 1200 liquid chromatography system coupled with a 6130 quadrupole mass spectrometer equipped with an ESI ion source (Agilent Technologies, Palo Alto, CA, USA). Analysis was performed at 40 °C on a Gemini C18 column (150 × 4.6 mm, 3 μm; Phenomenex, Torrance, CA, USA) with a mobile phase flow rate of 0.6 mL/min. Composition of eluent A was 0.1% (NH_4_)­(HCOO) in water; eluent B was 0.1% (NH_4_)(HCOO) and 8% water in acetonitrile, 0–3 min 5% B, 3–13 min gradient, and 13–20 min 100% B. The injection volume was 2 μL. The chromatographic profile was registered at 256 nm. The MSD operating parameters were as follows: positive ionization mode, scan spectra from *m*/*z* 120 to 1000, drying gas temperature 300 °C, nitrogen flow rate 12 L/min, nebulizer pressure 60 psi, and capillary voltage 4000 V.

Mass spectrometry coupled to gas chromatography (GC–MS) analysis was performed on an Agilent 6890 N-GC-5973 N-MSD system. The apparatus contained a 30 × 0.25 mm Restek Rtx-5SILMS column, which was covered by a 0.25 μm thick film on the inside. The temperature program of the column was as follows: 45 °C for 1 min, then 10 °C/min heating until 310 °C, then this temperature was held for 17 min. The temperature of the injector was 250 °C, and carrier gas was helium.

The structure of the capsaicinoid products was characterized by ^1^H NMR, as well as high-resolution mass spectroscopy (HR-MS). The ^1^H NMR spectra were obtained in CDCl_3_ or DMSO-*d*_6_ solution on a Bruker DRX-500 spectrometer operating at 500 and 125.7 MHz, respectively, with a chemical shift relative to tetramethylsilane (TMS), in parts per million (ppm) unit. Chemical shifts (δ) are reported in ppm upfield from TMS as an internal standard.

HR-MS measurements were performed on a Sciex TripleTOF 5600+ high resolution tandem mass spectrometer equipped with a DuoSpray ion source. Electrospray ionization was applied in positive ion detection mode. Samples were dissolved in acetonitrile and flow-injected into an acetonitrile/water 50:50 flow. The flow rate was 0.2 mL/min. The resolution of the mass spectrometer was 35,000.

The stoichiometry of capsaicinoids’ complexation with CDs was investigated by the Job’s method of continuous variation.^28^ Samples were prepared in D_2_O at neutral pH at a 25 °C temperature. The total molar concentration of the two components, capsaicinoid + CD, was kept constant at 0.2 mM, while the mole fraction of the capsaicinoids varied gradually in 0.1 unit steps from 0 to 1. Each sample contained 6% (v/v) deuterated methanol to ensure the desired solubility of capsaicinoids and also a minute amount of nondeuterated methanol as a chemical shift scale reference. ^1^H NMR chemical shifts of selected characteristic nonoverlapping resonances of the capsaicinoids were collected, and complexation-induced displacement values were calculated with respect to the chemical shift of the same resonance measured in the absence of CD. To construct Job’s plots, values were multiplied by the mole fraction of capsaicinoids and depicted as a function of the mole fraction of it.

To investigate the spatial arrangement of the host–guest complexes and identify the interacting molecular moieties, nuclear Overhauser effect (NOE) type experiments (2D NOEY and ROESY) were conducted. NOE is a manifestation of dipolar cross-relaxation between two nonequivalent nuclear spins that are close enough (<5 Å) through space. The NOE intensities are scaled with r-6, where r represents the mean distance between the protons. 2D spectra were acquired on the 400 MHz instrument, with mixing times of 350 ms using 16 scans on 1k × 512 data points.

Samples were prepared by adding 10 mM nonbuffered CD solutions to an excess of solid capsaicinoids to dissolve the maximum possible amount. The resulting suspensions were sonicated at room temperature for 10 min, stirred for 20 h, and then sonicated again for an additional 10 min. Finally, the suspensions were filtered into standard NMR tubes using 0.22 μm regenerated cellulose syringe filters.

### 2.3 Phase-Solubility Study

Phase-solubility tests were performed according to Higuchi and Connors.^29^ Phase-solubility diagrams demonstrate how the total drug solubility changes with an increasing CD concentration. When the drug–CD complex is soluble in aqueous media, A-type diagrams are formed. The A-type diagrams are usually formed when the used CDs have good solubility in aqueous media. There are three A-type diagrams: linear diagram (A_L_), positive deviation from linearity (A_P_), and negative deviation from linearity (A_N_). When the complex has limited solubility in the tested media, B-type diagrams are formed. The B-type diagrams are mostly formed when native CDs are used because these CDs have limited solubility in aqueous media. There are two B-type diagrams: the complex limited solubility (B_S_), and the complex is insoluble (B_i_).^30^ The phase-solubility diagrams are shown in Figure 1.

**Figure 1:**
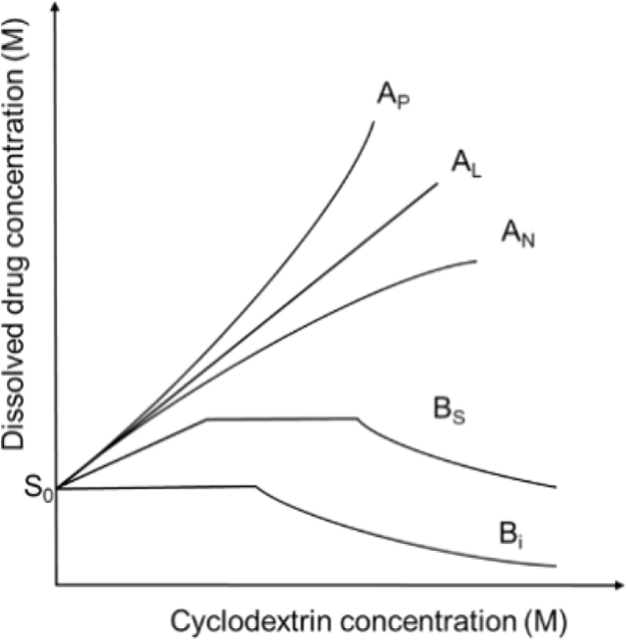
Phase-solubility diagrams according to Higuchi and Connors.^29^ Adapted with permission from Higuchi, T.; Connors, K. A. Phase-Solubility Techniques. *Adv. Anal. Chem. Instrum.* **1965,** *4,* 117. Copyright 1965 John Wiley & Sons.

The concentration of the tested CDs was in the range of 0–40 mM, and the guest molecules were added in excess to the CD solution; therefore, finally, suspensions were prepared. The suspensions were stirred with a magnetic stirrer at 25 °C for 24 h, filtered using 0.45 μm FilterBio PTFE Syringe filters (FilterBio Membrane Co., Nantong, China), and diluted 2-fold with purified water.

The concentration of the guest molecules in the filtered samples was determined on a Gemini NX-C18 50 × 4.6 mm, 110 Å (Phenomenex Inc., Torrance, CA, USA) analytical column by Agilent 1100 HPLC system equipped with a photodiode array detector. The utilized HPLC method was as follows: mobile phase-A: PW, mobile phase-B: ACN, flow rate: 1.0 mL/min, gradient elution: from 30% channel-B to 100% channel-B in 10 min, stop time: 10 min, equilibration time: 4 min, injection volume: 5.0 μL, column temperature: 30 °C, and detection at 222 nm.

The following eqs (eqs 1 and 2) were used to determine the complex apparent stability constant (*K*_s_) and the complexation efficacy (CE)

1 $$K_{s}=\frac{slope}{S_{0}\left(1-slope\right)}$$

2 $$CE=\frac{slope}{1-slope}$$

where *S*_0_ is the intrinsic solubility of a poorly soluble drug.

The binding strength between CD and the guest molecule is described by the apparent stability constant of the complex, but several different types of complexes can coexist in the solution (e.g., inclusion complexes, noninclusion complexes, aggregates of CDs, or CD–guest molecule complexes). Because of the different forms in the solution, the determination of CE can be very useful in the comparison of the solubilizing effect of CDs.^31^

## 3 Results and Discussion

### 3.1 Scaled-Up Synthesis of Capsaicinoids

As a preliminary result in our earlier research, we reported the first flow synthesis of capsaicin and its analogues through three optimized steps: (1) oxime formation of benzaldehydes, (2) reduction of oximes to benzylamines, and (3) *N*-acylation with activated carboxylic acids.^16^ Each step was first optimized individually (Table 1) and then integrated into a semicontinuous flow process, with a solvent exchange between the steps.

**Table 1:**
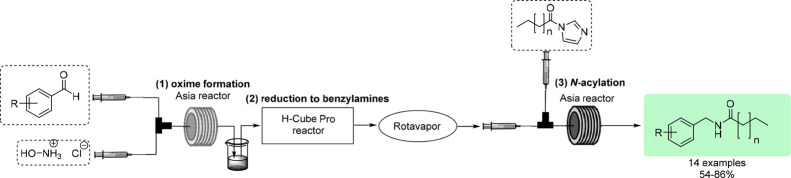
Optimal Conditions for Our Earlier Developed Flow Synthesis of Capsaicinoids^16^ ^,^ ^b^

reaction step	reagents/catalysts	optimal conditions	comments
oxime formation	benzaldehydes, hydroxylamine hydrochloride, NaOH	25 °C, 3 min, 0.67 mL/min, 1.2 equiv of reagent/base, MeOH	nearly full conversion, no need for heating
hydrogenation	oximes, Raney-Ni catalyst, MeOH/NH_3_	120 °C, 31 s, 1 bar H_2_, 1.56 mL/min, 1 M MeOH/NH3	high selectivity, in situ formation of H_2_
*N*-acylation	primary amines, CDI-activated^a^ carboxylic acids, 2-MeTHF/IPA	70 °C, 8 min, 7 bar, 0.25 mL/min, 0.1 M solution of amines, 1 equiv. activated acid	homogeneous mixtures, green solvent, good to high final yield

a 1,1′-Carbonyldiimidazole.

b Adapted with permission from Orosz, M. J.; Rávai, B.; Mátravölgyi, B.; Bálint, E. Flow Synthesis of Capsaicin and Capsaicinoid Analogues. *ACS Sustain. Chem. Eng.* **2024,** *12*, 7913. Copyright 2024 American Chemical Society.

From a green chemical perspective, our method significantly improves atom economy, reduces waste (lower E-factors), avoids hazardous reagents (e.g., thionyl chloride and hydrogen cylinders), and uses safer solvents (IPA and 2-MeTHF) as well as in situ hydrogen generation by a H-Cube Pro continuous flow hydrogenation reactor. The process complies with 7 of the 12 principles of green chemistry, including waste prevention, catalysis, safer solvents, renewable raw materials, and safer technology. Our group also developed low-cost 3D-printed polypropylene reactors to perform parts of the synthesis and therefore provide a cheaper and scalable alternative to expensive commercial flow systems. Overall, our work provides a safer, faster, more sustainable, and more efficient synthetic preparation of capsaicinoids compared with conventional batch syntheses. The comparison between previously reported batch methods and our flow synthesis, along with the calculations for the green metrics, can be found in the Supporting Information and Tables S1–S3.

Based on these previous experiments, in this study, our aim was to scale up and enhance the productivity of the flow synthesis of three main capsaicinoid derivatives, capsaicin (**6a**), dihydrocapsaicin (**6b**), and nonivamide (**6c**). Furthermore, we aimed to produce these compounds in sufficient quantities for the next phase of our research, in which their complexation behavior was investigated with α- and β-CD derivatives.

For this purpose, first, the substrate concentration had to be increased in the technology. In the first oxime formation reaction step (Scheme 1), the concentration of vanillin (**1**) was increased 10-fold. However, due to the insolubility of a higher amount of hydroxylamine hydrochloride (**2**) in methanol, water had to be introduced as a cosolvent. Fortunately, also in MeOH/H_2_O = 1:1, the oxime (**3**) formation proceeded practically quantitatively in a quick manner using a Syrris Asia flow reactor, with a yield of 98% and a productivity of up to 13.1 g/h.

**Scheme 1:**
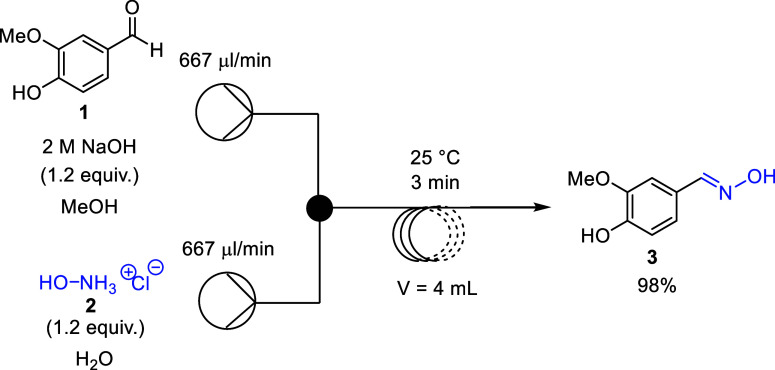
Scaled-Up Continuous Flow Synthesis of Vanillin Oxime (**3**)

Next, the scaled-up flow synthesis of the key intermediate, vanillylamine (**4**), was studied in the H-Cube Pro continuous hydrogenating reactor using the Raney nickel catalyst. During hydrogenation, ammonia additive was required to reduce the amount of byproduct (**5**) (Table 2). Thus, 25% NH_3_/H_2_O was used as an ammonia source. First, the reduction was attempted on the crude reaction mixture of oxime formation (Scheme 1) to study the possibility of combining the previous step and the reduction in a continuous flow system; however, the hydrogenation reactor became clogged due to the presence of inorganic salts. Consequently, the reaction mixture from the previous step had to be worked up, and the oxime was dissolved in H_2_O/MeOH mixture, and 25% NH_3_/H_2_O was added, thus 10 equiv of NH_3_ were present in the solution to achieve good selectivity for the primary amine. The flow rate of the vanillin oxime (**3**) solution was set to 1 mL/min. Initially, the effect of the temperature on conversion and selectivity was studied (Table 2). No reaction occurred up to 50 °C (Table 2/entries 1,2); however, from 75 °C the conversion was complete (Table 2/entries 3–7). Looking at the results, the next goal was to increase the selectivity of the reduction. It became clear that the selectivity decreased by increasing temperature (Table 2/entries 3 and 7), and thus, at 75 °C, the effect of H_2_ pressure was investigated. Better selectivity was observed at higher pressure (Table 2/entries 4–6), and the best result (60% GC yield) was obtained at 75 °C, using 41 bar H_2_ pressure (Table 2/entry 5).

**Table 2:**
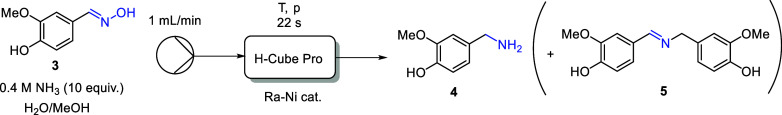
Optimization of the Scaled-Up Flow Synthesis of Vanillylamine (**4**)

entry	*T* (°C)	H_2_ pressure (bar)	conversion of 3 (%)^a^	ratio of 4 (%)^a^	ratio of 5 (%)^a^
1	25	2	0	0	0
2	50	2	0	0	0
3	75	2	100	51	49
4	75	21	100	52	48
5	75	41	100	60	40
6	75	61	100	58	42
7	100	2	100	41	59

a Determined by GC.

Finally, the scale-up of the *N*-acylation step, leading to the target capsaicinoids (**6a–c**), was investigated. This step could not be connected to the previous one, as the MeOH/H_2_O solvent immediately decomposed the acylating agents (**5a–c**). However, after workup and purification of vanillylamine (**4**), the *N*-acylation was also scaled up by 10-fold with conditions according to our previous experiments (70 °C, 8 min) (Scheme 2). After workup and purification, capsaicin (**6a**), dihydrocapsaicin (**6b**), and nonivamide (**6c**) were obtained with good to high yields and enhanced productivity of up to 3.3–4 g/h.

**Scheme 2:**
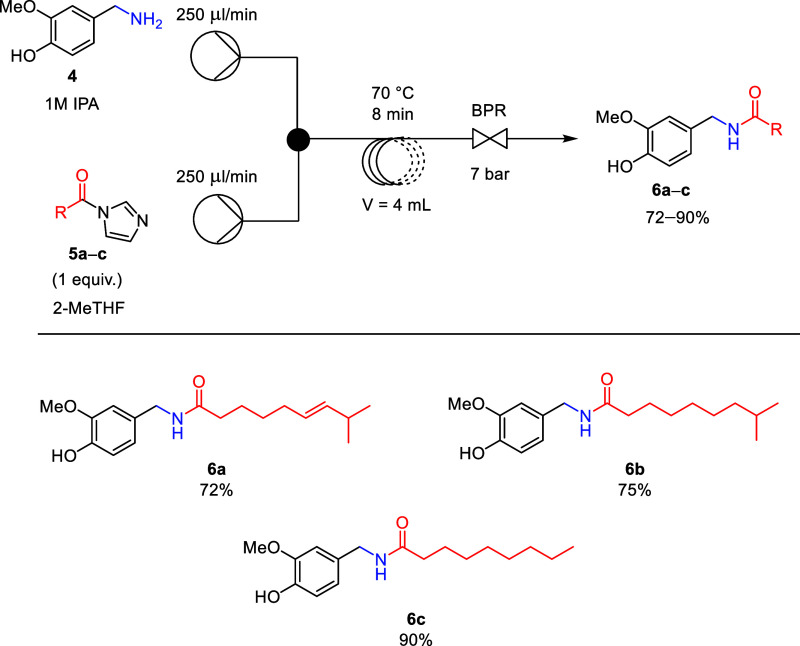
Scaled-Up Flow Synthesis of Capsaicinoids **(6a–c)**

### 3.2 Phase-Solubility Studies

The phase-solubility analysis is the most common method for determining the stoichiometry of the complex and the effect of CD-complexation on the water solubility of guest molecules. Usually, the stoichiometry of the drug–CD complexes is 1:1 (i.e., one drug molecule and one CD molecule form a complex).

During our research work, with the scaled-up synthesis in hand, the phase-solubility analysis of the three main types of capsaicinoids (**6a–c**) were studied with a wide range of CD derivatives, i.e., α-CD, HP-α-CD (average degree of substitution (DS) = 4.5), RAME-α-CD (average DS = 11.0), HP-β-CD (average DS = 4.5), HP-β-CD (average DS = 7.0), RAME-β-CD (average DS = 12.0), and SBE-β-CD (average DS = 6.5), where the DS is the average DS of the CD in each cases.

Overall, the choice to use only α- and β-CD and their substituted derivatives for the complexation of capsaicinoids is mainly determined by the size compatibility between the CD cavity and the guest molecules. They can easily provide optimal cavity diameters for the inclusion of capsaicinoids: α-CD (approximately 0.47–0.53 nm) and β-CD (about 0.60–0.65 nm), which closely match the molecular dimensions of capsaicin and its analogues (1.0–1.2 nm in length), resulting in efficient host–guest interactions and well-defined complexation behavior.^32,33^ In contrast, γ-CD has a much larger cavity (0.75–0.83 nm), which is less suitable for stabilizing relatively small, linear molecules, which could result in weaker and less specific inclusion complexes. γ-CD is instead better suited for encapsulating significantly bulkier or sterane-core compounds and is rarely employed in food industry applications for this purpose. Moreover, γ-CD can self-assemble and form aggregates, resulting in nano- and microparticles; though the majority is present in monomeric form, some fraction may exist as transient clusters or aggregates. This aggregation behavior can also influence its practical use in formulations.

Based on the phase-solubility diagrams (Figures 2–4), most of the curves obtained in this study are linear (A_L_-type), and there is only one nonlinear curve (nonivamide (**6c**)—α-CD), which shows negative deviation from linearity (A_N_-type) (Figure 4). A_L_-type phase-solubility diagrams are characterized by a linear increase in the equilibrium concentration of dissolved drug as the concentration of CD, while A_N_-type phase-solubility diagrams show a negative deviation from linearity, which suggests the formation of higher-order complexes, self-association, or changes in stoichiometry, often reducing the solubility enhancement at higher CD concentrations.^29^

**Figure 2:**
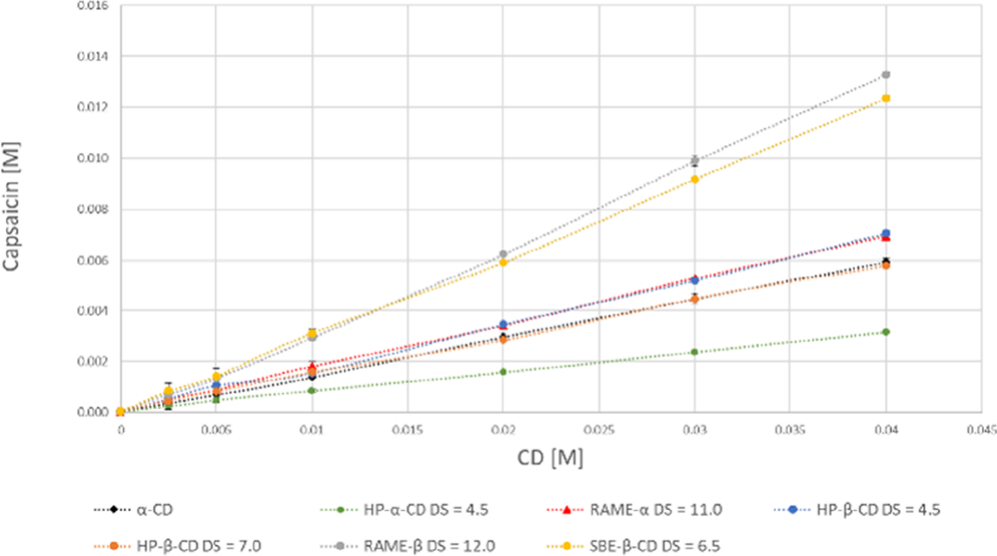
Phase-solubility curves of capsaicin (**6a**) and the tested CDs.

**3:**
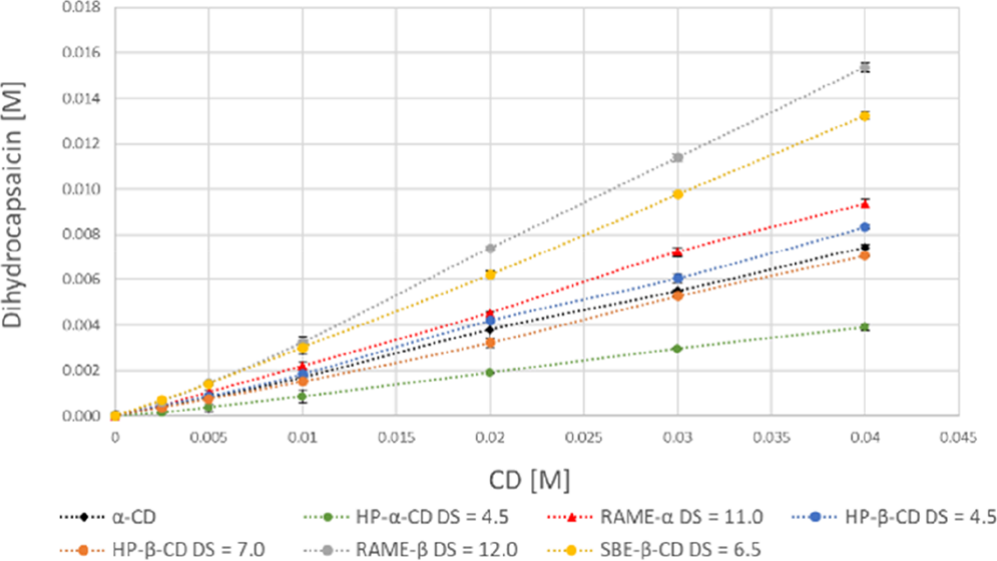
Phase-solubility curves of dihydrocapsaicin (**6b**) and the tested CDs.

**Figure 4:**
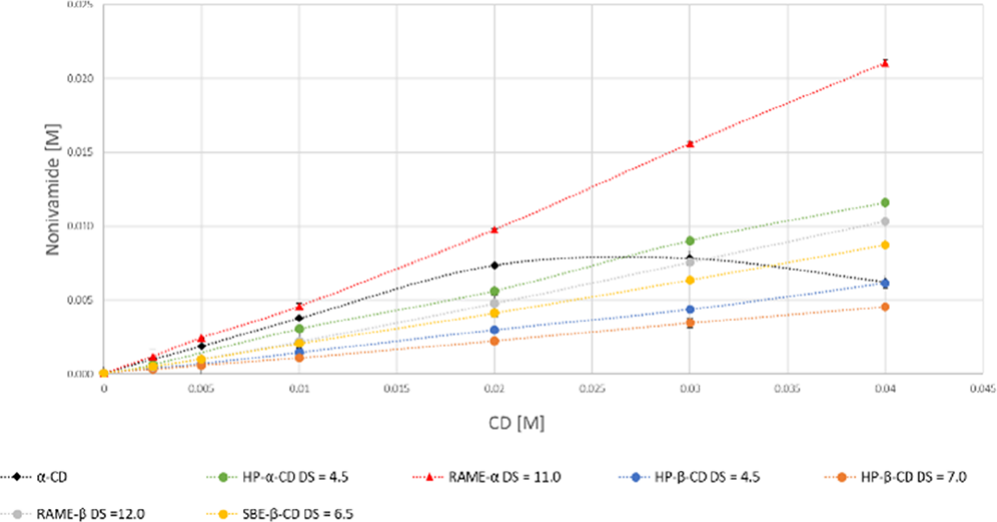
Phase-solubility curves of nonivamide (**6c**) and the tested CDs.

The phase-solubility curves of capsaicin (**6a**), dihydrocapsaicin (**6b**), and nonivamide (**6c**) with the tested CDs are shown in Figures 2–4. The calculated apparent stability constants and calculated CE values are also given in Tables 3–5.

**Table 3:** Stability Constants (*K*_s_), Complexation Efficiencies, and Solubility Enhancement (*S*_max_/*S*_0_) for Complexes of Capsaicin (**6a**) with the Tested CDs in Water at 25 °C, after 24 h (Number of Measurements = 3; SE = Standard Error of the Mean)

	α-CD	HP-α-CD (DS = 4.5)	RAME-α-CD (DS = 11)	HP-β-CD (DS = 4.5)	HP-β-CD (DS = 7.0)	RAME-β-CD (DS = 12)	SBE-β-CD (DS = 4.5)
*S*_0_ (average)	0.00004	0.00004	0.00004	0.00004	0.00004	0.00004	0.00004
slope (average)	0.1485	0.077	0.1724	0.1733	0.1430	0.3343	0.3067
*K*_s_ [M^–1^]	4360 ± 110	2090 ± 30	5200 ± 80	5240 ± 170	4170 ± 40	12,550 ± 240	11,060 ± 220
CE [%]	0.174 ± 0.005	0.083 ± 0.01	0.208 ± 0.003	0.2096 ± 0.0003	0.167 ± 0.001	0.502 ± 0.003	0.442 ± 0.005
*S*_max_/*S*_0_	142 ± 8	81 ± 3	174 ± 5	169 ± 9	145 ± 5	332 ± 15	309 ± 9

**4:** Stability Constants (*K*_s_), Complexation Efficiencies, and Solubility Enhancement (*S*_max_/*S*_0_) for Complexes of Dihydrocapsaicin (**6b**) with the Tested CDs in Water at 25 °C, after 24 h (Number of Measurements = 3; SE = Standard Error of the Mean)

	α-CD	HP-α-CD (DS = 4.5)	RAME-α-CD (DS = 11)	HP-β-CD (DS = 4.5)	HP-β-CD (DS = 7.0)	RAME-β-CD (DS = 12)	SBE-β-CD (DS = 4.5)
*S*_0_ (average)	0.00001	0.00001	0.00001	0.00001	0.00001	0.00001	0.00001
slope (average)	0.1871	0.0994	0.2382	0.2092	0.1776	0.3903	0.3318
*K*_s_ [M^–1^]	16,750 ± 250	7750 ± 80	22,100 ± 130	18,570 ± 320	15,660 ± 190	45,130 ± 310	34,650 ± 290
CE [%]	0.237 ± 0.004	0.110 ± 0.001	0.315 ± 0.002	0.265 ± 0.005	0.223 ± 0.003	0.643 ± 0.002	0.494 ± 0.003
*S*_max_/*S*_0_	541 ± 8	273 ± 3	662 ± 4	586 ± 10	512 ± 6	1083 ± 15	924 ± 8

**Table 5:** Stability Constants (*K*_s_), Complexation Efficiencies, and Solubility Enhancement (*S*_max_/*S*_0_) for Complexes of Nonivamid (**6c**) with the Tested CDs in Water at 25 °C, after 24 h (Number of Measurements = 3; SE = Standard Error of the Mean)^a^

	α-CD	HP-α-CD (DS = 4.5)	RAME-α-CD (DS = 11)	HP-β-CD (DS = 4.5)	HP-β-CD (DS = 7.0)	RAME-β-CD (DS = 12)	SBE-β-CD (DS = 4.5)
*S*_0_ (average)	0.00007	0.00007	0.00007	0.00007	0.00007	0.00007	0.00007
slope (average)	0.3644	0.292	0.5253	0.1509	0.1129	0.2591	0.2158
*K*_s_ [M^–1^]	7740 ± 80	5570 ± 80	14,950 ± 250	2400 ± 20	1780 ± 20	4720 ± 110	3720 ± 100
CE [%]	0.573 ± 0.004	0.412 ± 0.007	1.106 ± 0.01	0.178 ± 0.001	0.127 ± 0.002	0.349 ± 0.005	0.275 ± 0.009
*S*_max_/*S*_0_	–	156 ± 5	285 ± 11	83 ± 3	62 ± 4	140 ± 9	118 ± 10

a Only the linear part of the curve (0–0.02 M α-CD) was used in the determination of the *K*_s_, CE, and *S*_max_/*S*_0_.

The *K*_s_ and CE values are consistent with increasing the water solubility of the guest molecules. The results show that the randomly hydroxypropylated CDs (HP-α-CD and HP-β-CD) increased the water solubility of guest molecules (**6a–c**) the least, while the highest solubility increases were obtained in all cases by the randomly methylated CD derivatives. RAME-β-CD improved the water solubility the most in the case of capsaicin (**6a**) and dihydrocapsaicin (**6b**), while RAME-α-CD enhanced the water solubility of nonivamide (**6c**). After RAME-β-CD, SBE-β-CD increased the water solubility of capsaicin (**6a**) and dihydrocapsaicin (**6b**) the most. However, in the case of nonivamide (**6c**), α-CD had a better effect on the increasing solubility after RAME-α-CD.

In the case of the studied complexes, we have uniquely observed an A_N_-type phase-solubility curve for the nonivamide (**6c**)—α-CD complex. In this case, a very fast formation of precipitation was observed in the filtered sample; thus, after filtration, the samples were filtered again before the HPLC measurement. In the determination of the stability constant, only the linear part of the curve was used.

Overall, for capsaicin (**6a**) and dihydrocapsaicin (**6b**), the β-cavity CDs, while for nonivamide (**6c**), the α-cavity CDs increased the water solubility the most, which is influenced by the structure of the guest molecule. The cavity size of α-CD is appropriate for linear carbon chains, while the larger cavity of β-CD enables complexing larger molecular fragments, such as a benzene ring. The tested capsaicinoids (**6a–c**) contain a 9-membered aliphatic chain, but capsaicin (**6a**) and dihydrocapsaicin (**6b**) have a branch at the end of the carbon chain, so these chains are presumably not complexed with α-CD. However, in the case of nonivamide (**6c**), the complexation with α-CD is favored, presumably because there is no branching at the end of the carbon chain.

It is important to note that the intrinsic solubility of the guest molecules has a very large effect on the calculated stability constants. Based on our measurements, dihydrocapsaicin (**6b**) has the lowest intrinsic water solubility; thus, we obtained the highest apparent stability constants for this guest molecule (**6b**) among the three tested capsaicinoids (**6a–c**). In this case, it is advisable to utilize the CE value, which is independent of the intrinsic solubility of the guest molecule. The CE values are consistent with the K_s_ values; however, the highest CE value was obtained for nonivamide (**6c**–RAME-α-CD complex), which may indicate a significant stability of this complex.

Phase-solubility studies of capsaicinoids (**6a–c**) were also performed with the native β-CD in the range of 0–15 mM of CD. The phase-solubility curves of capsaicin (**6a**), dihydrocapsaicin (**6b**), and nonivamide (**6c**) with β-CD are shown in Figure 5.

**Figure 5:**
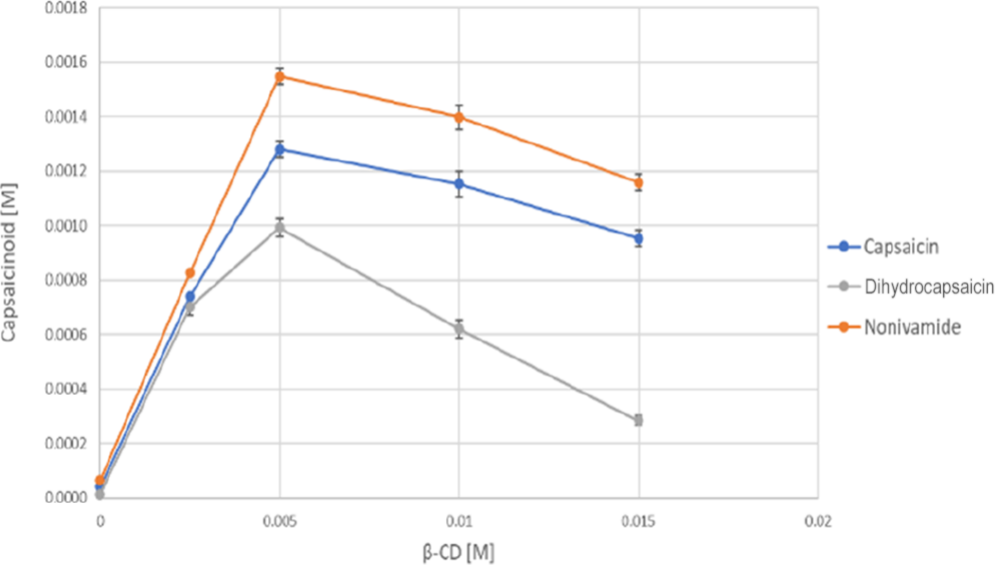
Phase-solubility curves of capsaicin (**6a**), dihydrocapsaicin (**6b**), and nonivamide (**6c**) with β-CD.

In the case of β-CD, the phase-solubility curves are B_S_-type, and the solubility-enhancing effect of β-CD is also very low. B-type phase-solubility diagrams (e.g., B_S_-type) display a profile where the drug concentration initially rises in the presence of a CD; however, it finally reaches a plateau or even decreases, indicating limited aqueous solubility of the complex and possible precipitation of insoluble complexes at high CD levels.^29^ For these reasons, no further investigations were carried out with this type of CD, but the *K*_s_, CE, and *S*_max_/*S*_0_ values were also calculated from the phase solubility curves (Table 6). In all cases, only the linear part of the curves was used (0–0.005 M β-CD). Based on the results, β-CD forms the most stable complex with dihydrocapsaicin (**6b**); however, the CE values are also worth considering. In the case of the calculation of CE, the solubility of capsaicinoids without CD is not included in the formula; thus, CE better illustrates the solubilizing effect of CDs. In terms of CE results, nonivamid (**6c**) dissolved at the highest concentration of the three capsaicinoids, at the concentration of 0.005 M β-CD. This result is consistent with the phase solubility curves.

**Table 6:** Stability Constants (*K*_s_), Complexation Efficiencies, and Solubility Enhancement (*S*_max_/*S*_0_) for Complexes of the Tested Capsaicinoids with β-CD in Water at 25 °C, after 24 h (Number of Measurements = 3; SE = Standard Error of the Mean)

	capsaicin (**6a**)	dihydrocapsaicin (**6b**)	nonivamide (**6c**)
*S*_0_ (average)	0.00004	0.00001	0.00007
slope (average)	0.2477	0.1883	0.2909
*K*_s_ [M^–1^]	8230 ± 10	23,200 ± 950	5860 ± 160
CE [%]	0.3293 ± 0.0004	0.232 ± 0.009	0.410 ± 0.012
*S*_max_/*S*_0_	30.7 ± 0.7	67.3 ± 2.3	20.8 ± 1.7

As a summary, in literature, capsaicinoids are described as very poorly water-soluble compounds, with solubilities typically in the range of 10^–5^–10^–4^ M. For instance, capsaicin (**6a**) is generally reported as “insoluble” or <0.02 mg/mL in water, while dihydrocapsaicin (**6b**) exhibits an even lower solubility of around 0.003 mg/mL, and nonivamide (**6c**) is also below 0.02 mg/mL.^34–36^

In contrast, in our study, our inclusion complexation with CD derivatives increased aqueous solubility by 2 to 3 orders of magnitude. Specifically, capsaicin (**6a**) reached ∼4.0 mg/mL with RAME-β-CD (≈332-fold increase), dihydrocapsaicin (**6b**) ∼3.2 mg/mL with RAME-β-CD (≈1083-fold increase), and nonivamide (**6c**) ∼6.0 mg/mL with RAME-α-CD (≈285-fold increase). Therefore, while free capsaicinoids are restricted to the low μg/mL range in water, their CD inclusion complexes reach the milligram/milliliter range, a solubility level that is pharmaceutically and technologically highly significant. Moreover, the inclusion complexation also reduced the irritant character of the free capsaicinoid compounds as well, resulting in formulations formed in a denser, nondusty solid state rather than in a fine, airborne powder.

### 3.3 Nuclear Magnetic Resonance Studies

To confirm the complexation stoichiometry, an independent technique, NMR spectroscopy, was used, as it provides atomic-level insight into the intermolecular interactions between host and guest molecules. Although there is valid criticism regarding the limitations of Job’s method for characterizing equilibrium systems, as supported by our previous study, these limitations are predominantly for cases more complex than simple 1:1 interactions.^37–39^ Therefore, Job’s method remains a useful and reliable approach for determining the stoichiometry of 1:1 complexes, provided that the data are interpreted with due care, especially in the context of supramolecular chemistry.

Job’s plots were constructed using well-resolved NMR signals of the capsaicinoids (**6a–c**), such as the methyl proton resonances at the terminal end of the aliphatic side chain. The complexation-induced chemical shift changes observed for the capsaicin (**6a**)-α-CD system were almost negligible: neither the aromatic nor the aliphatic proton signals exhibited shifts exceeding the measurement error (∼2 ppb). However, subtle chemical shift perturbations were detected for the aliphatic methyl resonances. The relatively flat Job’s plot curves shown in Figure 6A indicate that, under experimental conditions, native α-CD does not induce a sufficiently measurable interaction to allow for quantifiable stoichiometric analysis. Subsequently, RAME-α-CD was applied to explore the molecular interactions with the capsaicinoids (**6a–c**) studied, since methylation of hydroxyl groups on the α-CD rim reduces the polarity of the inner cavity, creating a more hydrophobic environment. This is more in line with the nonpolar, lipophilic character of capsaicin (**6a**), especially its long aliphatic chain, which leads to more favorable van der Waals and hydrophobic interactions. The capsaicin (**6a**)-RAME-α-CD system still showed very limited to no chemical shift changes in the aromatic and aliphatic regions, except for the methyl resonances. The Job’s plot curve shown for this resonance (see Figure 6B) shows a maximum at 0.5, indicating the exclusive formation of the 1:1 complex. Figure 6C,D confirms the same stoichiometry for both dihydrocapsaicin (**6b**) and nonivamide (**6c**). All the maxima at 0.5 suggested the sole formation of the complexes with a stoichiometry of 1:1 between all three studied capsaicinoids (**6a–c**) and CDs.

**Figure 6:**
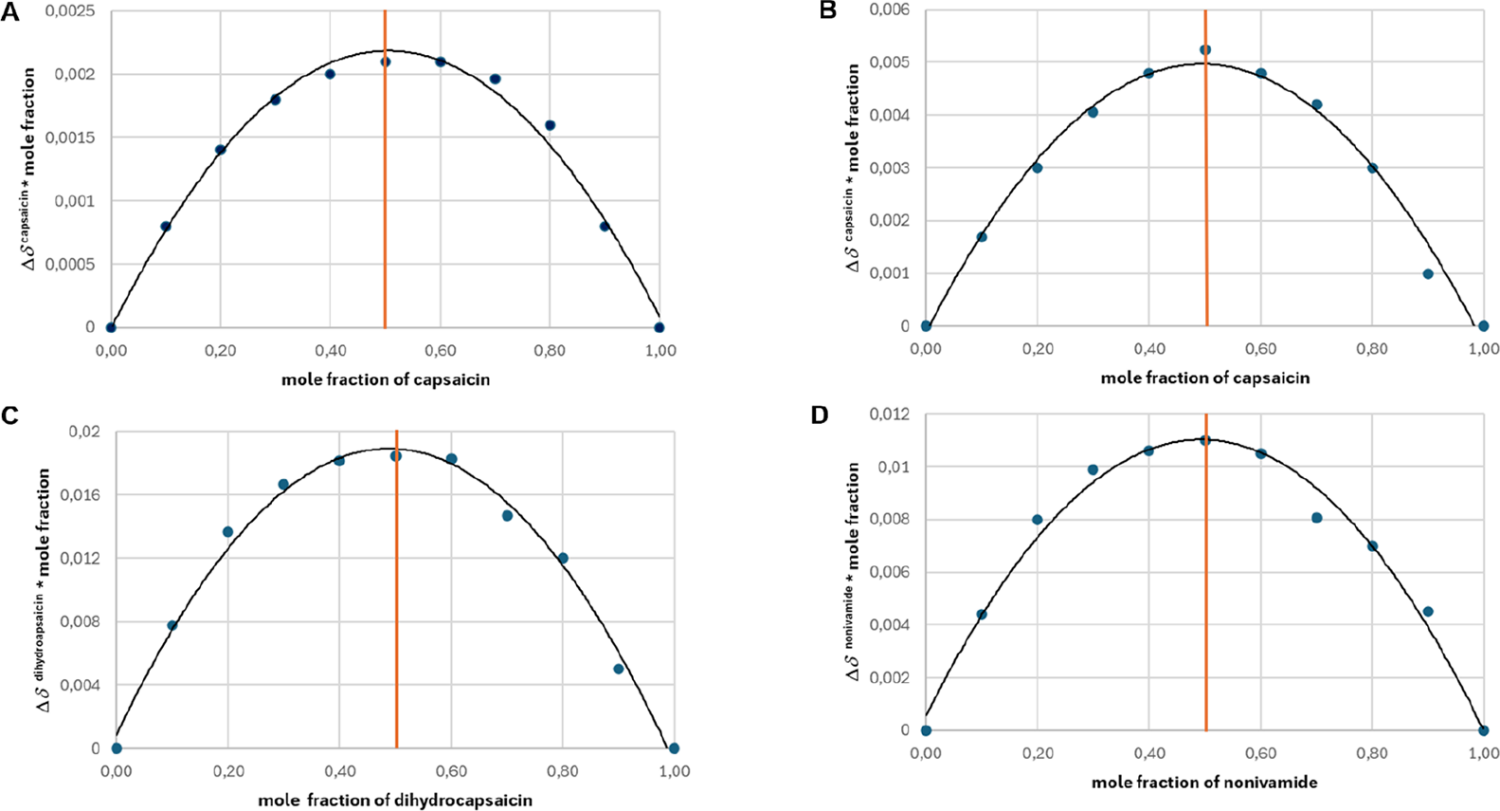
Job’s plots of capsaicinoid–CD systems, based on NMR chemical shift changes of the terminal methyl group of the capsaicinoid side chain. (**A**) Capsaicin (**6a**)−α-CD, (**B**) Capsaicin (**6a**)–RAME-α-CD, (**C**) dihydrocapsaicin (**6b**)–RAME-α-CD, and (**D**) nonivamide (**6c**)–RAME-α-CD.

Following the confirmation of 1:1 stoichiometries for all host–guest complexes using Job’s plots, we turned to detailed 2D ROESY NMR investigations to elucidate the spatial arrangement and specific interactions between the CDs and the studied capsaicinoid derivatives (**6a–c**). Special attention was given to how minor structural differences in the side chains of capsaicin (**6a**), dihydrocapsaicin (**6b**), and nonivamide (**6c**) influence their complexation behavior with native α-CD, HP-α-CD, and RAME-α-CD. Interestingly, in contrast to some previous reports in the literature, our ROESY spectra did not provide evidence for host–guest interactions of aromatic moieties of capsaicinoids (**6a–c**) and the CD cavity. This absence was consistent for all three CDs investigated. This observation aligns with the well-established preference of α-CD for binding linear aliphatic chains rather than aromatic systems.

To better understand the role of side chain architecture, we first focused on complexation with native α-CD, which, as a single chemical entity, CD, offers a straightforward interpretation due to the lack of substitution heterogeneity. Among the three capsaicinoids (**6a–c**), capsaicin (**6a**) exhibited the weakest interaction with α-CD. The only detectable ROESY cross-peak was between the terminal methyl group of the branched nonivamide side chain and the H3 proton of the CD cavity, indicating limited inclusion. The directionality of inclusion appears to be from the secondary (wider) rim of α-CD, which only encapsulates the more sterically accessible, conformationally restricted end of the alkyl chain.

In contrast, for dihydrocapsaicin (**6b**) with a fully saturated side chain, ROESY spectra revealed significantly more intense cross-peaks between the terminal methyl groups and the H3 protons of α-CD. In addition, weaker but detectable correlations between the adjacent methylene groups and the same H3 protons suggest a deeper and more flexible passage of the alkyl chain through the CD cavity. This probably results from the increased conformational freedom provided by the saturated chain, which facilitates sliding and dynamic interactions within the hydrophobic cavity.

In the case of nonivamide (**6c**), which lacks bulky branching at the end of its aliphatic chain, the ROESY spectra demonstrated the most extensive interaction pattern. Strong cross-peaks were observed not only for the terminal methyl, but also for several methylene units, including those directly adjacent to the amide functionality. These results support the hypothesis that the entire aliphatic chain of the nonivamide is located in the α-CD cavity, further confirming the structure-affinity trend observed in phase-solubility studies.

These NMR results confirm that the α-CD inclusion mechanism is highly sensitive to subtle variations in the aliphatic side chain structure of capsaicinoids (**6a–c**). The branching and unsaturation significantly reduce the depth and strength of inclusion, while linear, flexible chains promote deeper, more stable complex formation. These insights provide a rational basis for the observed differences in binding strength and solubility enhancement.

To further explore the effects of cavity modification, RAME-α-CD was also investigated. Random methylation results in substitution at both rims of the CD, effectively extending and slightly widening the cavity. However, this modification creates structural microheterogeneity, as the methylation is not site selective and generates a mixture of isomeric CDs. This microheterogeneity significantly complicates atomic-level structural analysis using liquid-state NMR, since the superposition of multiple isomers leads to broader, overlapping signals and attenuated cross-peak resolution in 2D spectra.

Nevertheless, RAME-α-CD allowed the use of higher capsaicinoid concentrations due to its enhanced solubility properties. Despite the spectral complexity, consistent patterns were observed in the ROESY measurements. In the case of capsaicin (**6a**), only very weak cross-peaks were detected between the terminal methyl group of the side chain and the protons of the CD cavity, again indicating the weakest inclusion among the three derivatives. For dihydrocapsaicin (**6b**), weak cross-peaks were observed between the terminal methyl and some adjacent methylene protons, although they were close to the noise level. This does not necessarily indicate a weak interaction; rather, the diffuse and poorly resolved signals are likely due to dynamic averaging and spectral broadening caused by CD microheterogeneity.

In contrast, nonivamide (**6c**) once again showed the most pronounced interaction with RAME-α-CD. In addition to the terminal methyl, ROESY spectra showed more intense and better-defined cross-peaks with multiple methylene units, suggesting that the entire aliphatic chain is immersed in the modified cavity. These observations are in excellent agreement with the trends found for native α-CD and further emphasize the high affinity of nonivamide for α-cavity CDs, regardless of the substitution pattern.

In summary, the ROESY studies underline that structural differences in capsaicinoid side chains and the physicochemical nature of the CD cavity have a significant influence on complexation behavior. While native α-CD enables cleaner spectral interpretation, RAME-α-CD offers improved solubility and affinity at the expense of spectral resolution. Nevertheless, both systems reveal a consistent interaction pattern: nonivamide (**6c**) > dihydrocapsaicin (**6b**) > capsaicin (**6a**).

## 4 Conclusion

The first pilot-scale flow chemical synthesis of capsaicin (**6a**), dihydrocapsaicin (**6b**), and nonivamide (**6c**) was developed using the Syrris Asia heatable reactor and H-Cube Pro continuous flow hydrogenation reactor through three consecutive reaction steps, such as oxime formation, hydrogenation, and *N*-acylation. Good to high yields and productivity of up to 3.3–4 g/h have been achieved in the synthetic production of capsaicinoids (**6a–c**). Detailed inclusion complexation studies were performed with three guest molecules (**6a–c**) and seven α- and β-CD derivatives to investigate the effect of CD complexation on the water solubility of capsaicinoids (**6a–c**). During the phase-solubility analysis, A_L_-and A_N_-type phase-solubility diagrams were obtained, and K_S_ and CE data were also calculated. While HP-α-CD and HP-β-CD increased the water solubility of the capsaicinoids (**6a–c**) the least, the highest solubility increase was obtained with the randomly methylated CD derivatives. Overall, β-cavity CDs increased water solubility the most for capsaicin (**6a**) and dihydrocapsaicin (**6b**), while α-cavity CDs increased water solubility the most for nonivamide (**6c**). The exact structure–effect correlations were confirmed by extensive NMR studies. Job’s plot analyses confirmed the formation of an exclusive 1:1 inclusion complex for all three capsaicinoids (**6a–c**) with both native and methylated α-CDs. Furthermore, 2D ROESY NMR measurements revealed that the depth and strength of inclusion are controlled by the structural features of the aliphatic side chains: linear and conformationally unrestricted nonivamide (**6c**) showed the most extensive inclusion, while branched or unsaturated side chains (capsaicin (**6a**) and dihydrocapsaicin (**6b**)) resulted in weaker interactions. These results provide direct atomic-level evidence of host–guest interaction patterns and provide a molecular explanation for the observed differences in solubility enhancement. The increased aqueous solubility and reduced pungency achieved by CD complexation may extend the use of capsaicinoids as pharmaceutical additives, natural preservatives, flavor modulators, and pesticides.

## Supplementary Material



## Notes

Additional references ( ^16^ and^40–42^ ) (PDF)
