# Supplementary material for: Pilot-Scale Continuous Flow Synthesis of Capsaicinoids and Their Formulation with Cyclodextrins
Source: ACS Omega. 2026 Jan 9;11(3):4570–80. doi: 10.1021/acsomega.5c10910 (PMC12854497; doi:10.1021/acsomega.5c10910)
Supplement: Supplementary file 1 [file ao5c10910_si_001.pdf]

Supporting Information

for

Pilot-scale Continuous Flow Synthesis of Capsaicinoids and their Formulation with Cyclodextrins

Bettina Rávai,[a,b] Dóra V. Ujj,[a] Máté J. Orosz,[a] Ecaterina Revenco,[a] Szabolcs Béni,[c] Ádám Tajti[d], and Erika Bálint[a]*

[a]*Department of Organic Chemistry and Technology, Faculty of Chemical Technology and Biotechnology, Budapest University of Technology and Economics, Műegyetem rkp. 3., H-1111 Budapest, Hungary*

[b]*CycloLab Cyclodextrin Research and Development Ltd., Illatos út 7., H-1097 Budapest, Hungary*

[c]*Department of Analytical Chemistry, Faculty of Science, Eötvös Loránd University, Pázmány Péter sétány 1/a., H-1117, Budapest, Hungary*

[d]*Bálint Analitika Ltd., Kondorfa u. 6., H-1116 Budapest, Hungary*

*Corresponding author: Erika Bálint, balint.erika@vbk.bme.hu

Table of Contents

1. Comparison of previous batch methods and our flow synthesis 3

2. Green metrics calculations 3

3. General procedures 4

3.1. General procedure for the flow synthesis of vanillin oxime (3) 4

3.2. General procedure for the flow synthesis of vanillylamine (4) 5

3.3. General procedure for the preparation of CDI-activated long-chain carboxylic acids (5a-c) 5

3.4. General procedure for the flow synthesis of capsaicin (6a), dihydrocapsaicin (6b) and nonivamide (6c) 6

4. ^{1}H NMR analysis for Job's plot method..7

5. ROESY analysis 11

6. References 14

1. Comparison of previous batch methods and our flow synthesis

Table S1. Comparison of earlier batch protocols with our flow-based synthesis

	reaction step	yield (%)	T (°C)	t or τ (min)	AE (%)	E-factor[a]	ref.
**Batch methods**	Oxime formation	76	100	10	55	2.24	S1
	Reduction	74	10–15	180	37	13.8	S1
	N-acylation	53[b]	25–35	240	69	4.22	S2
		71[c]	25	480	57	2.60	S3
**Our flow synthesis**	Oxime formation	95	25	3	64	0.78	
	Reduction	91	120	0.5	89	1.66	S4
	N-acylation	60[b]	70	8	63	1.71	
		77[c]	70	8	62	1.20	

[a]If solvents are reused. [b]The product is capsaicin (**6a**). [c]The product is nonivamide (**6c**).

2. Green metrics calculations

The methodology for calculating the green metrics (atom economy and E-factor) presented in Table S2 is illustrated using the oxime formation step, comparing Shafi and coworkers' batch method[S1] with our earlier reported flow synthesis[S4], representing in Table S3. In determining the E-factors, we assumed solvent recycling, and therefore only the masses of the reagents and product were considered in the calculations.

Table S2. Synthesis of vanillin oxime (3) according to *Shafi* et. al.

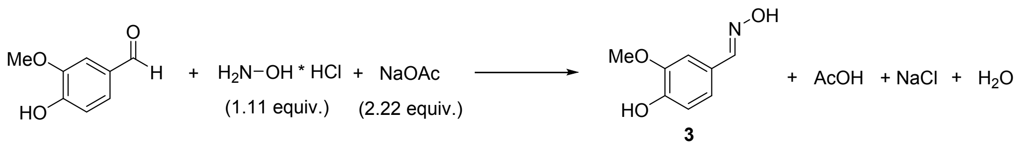

3
76%

	Vanillin	Hydroxylamine hydrochloride	Sodium acetate	Vanillin oxime (**3**)
Molar mass (g/mol)	152.15	69.49	82.03	167.16
Mass (g)	1.98	1.00	2.36	1.65
Equivalents	1	1.11	2.22	–
Moles (mmol)	13.0	14.4	28.8	9.85

Table S3. Synthesis of vanillin oxime (3) in this paper

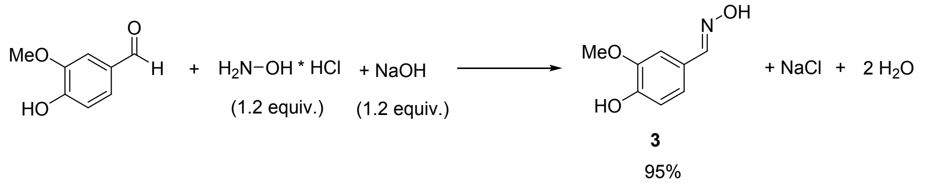

	Vanillin	Hydroxylamine hydrochloride	Sodium hydroxide	Vanillin oxime (**3**)
Molar mass (g/mol)	152.15	69.49	40.00	167.16
Mass (g)	0.152	0.083	0.048	0.159
Equivalents	1	1.2	1.2	–
Moles (mmol)	1	1.2	1.2	0.95

Atom-economy

AE_{batch} =

$$\frac{M(vanillin\ oxime)}{M(vanillin) + M(hydroxylamine * HCl) + M(NaOAc)} * 100\% = \frac{167.16}{152.15 + 69.49 + 82.03} * 100\% = \mathbf{55}\%$$

AE_{flow} =

$$\frac{M(vanillin\ oxime)}{M(vanillin) + M(hydroxylamine * HCl) + M(NaOH)} * 100\% = \frac{167.16}{152.15 + 69.49 + 40.00} * 100\% = \mathbf{64}\%$$

E-factor

E_{batch} =

$$\frac{m\ (waste)}{m(vanillin\ oxime)} = \frac{m(starting\ materials) - m(vanillin\ oxime)}{m(vanillin\ oxime)} = \frac{1.98 + 1.00 + 2.36 - 1.65}{1.65} = \mathbf{2.24}$$

E_{flow} =

$$\frac{m\ (waste)}{m(vanillin\ oxime)} = \frac{m(starting\ materials) - m(vanillin\ oxime)}{m(vanillin\ oxime)} = \frac{0.152 + 0.083 + 0.048 - 0.159}{0.159} = \mathbf{0.78}$$

3. General procedures

*3.1. General procedure for the flow synthesis of vanillin oxime (**3**)*

For the flow synthesis of vanillin oxime, a 2 M solution of vanillin (10 mmol) was prepared in a vial, in methanol (5 mL), then 1.2 equivalents of NaOH (0.480 g) was added to the solution (Solution A). In another vial, 1.2 equivalents (0.834 g) of hydroxylamine

hydrochloride was dissolved in distilled water (5 mL) (Solution B). First, the Syrris Asia® system was washed with $MeOH/H_2O$ 1:1 mixture at a flow rate of 2 mL/min for 6 minutes. Next, Solution A and B were pumped through the flow system, both at 0.667 mL/min flow rate. The received solution of the oxime was evaporated *in vacuo*, the remaining solid was washed with ethyl acetate (2 x 10 mL) and filtered. The filtrate was then evaporated in vacuo, yielding the pure oxime. The product was analysed by GC-MS.

*4-hydroxy-3-methoxybenzaldehyde oxime (**3**)*

White solid, yield: 98% (1.64 g), productivity: 13.1 g/h. GC-MS (m/z): 167 [M^+]

*3.2. General procedure for the flow synthesis of vanillylamine (**4**)*

First, an 0.4 M stock solution from pure vanillin oxime was prepared, by dissolving the substrate (10 mmol) in a flask, in $MeOH/H_2O$ = 1:1 (25 ml), containing 7.5 ml of 25% NH_3/H_2O. Next, the H-Cube Pro® hydrogenating reactor was washed with $MeOH/H_2O$ = 1:1 at a flow rate of 2 mL/min for 5 minutes using a Knauer Azura P2.1S HPLC pump. After that, the stock solution was pumped through the H-Cube Pro® system at a flow rate of 1 mL/min. The received solution was evaporated *in vacuo*, and the crude product was purified on a thin layer of silica (10 g, EtOAc eluent). The product was analysed by GC-MS.

*4-(Aminomethyl)-2-methoxyphenol (**4**)*

Light-yellow solid, yield: 56% (0.86 g), productivity: 2.06 g/h. GC-MS (m/z): 153 [M^+]

*3.3. General procedure for the preparation of CDI-activated long-chain carboxylic acids (**5a-c**)*

A 1 M solution of the corresponding long-chain carboxylic acid (5.0 mmol, obtained from Fluorochem Ltd. and Combi Blocks Ltd.) was prepared in 5 mL of absolute 2-MeTHF in a 25 mL round bottom flask with a magnetic stirrer. Next, 1.15 equivalent (0.93 g) of CDI was added at once during stirring, and a bubbler filled with toluene was placed on the flask. The reaction was monitored via the bubbler and GC-MS, however, after no more bubbling was observed at the bubbler, the conversion of the carboxylic acid was complete (depending on the substrate, in 30–90 minutes). The activated carboxylic acid derivatives were not isolated and were further used in their 2-MeTHF solution without any purification.

*3.4. General procedure for the flow synthesis of capsaicin (**6a**), dihydrocapsaicin (**6b**) and nonivamide (**6c**)*

For the flow synthesis of capsaicinoids, a 1 M solution of capsaicin (**6a**) or dihydrocapsaicin (**6b**) or nonivamide (**6c**) (5 mmol) was prepared in a vial, in isopropyl alcohol (5 ml) (Solution A). In another vial, 1 equivalent of freshly prepared CDI-activated 8-methylnon-6-enoic acid (**5a**) or 8-methylnonanoic acid (**5b**) or nonanoic acid (**5c**) (5 mmol) in 2-methyltetrahydrofuran (5 ml) was placed (Solution B). First, the Syrris Asia® system was washed with isopropyl alcohol at a flow rate of 2 mL/min for 6 minutes, and a 7 bar back-pressure regulator was connected to the exit of the reactor. Then, Solution A and B were pumped through the flow system, both at 0.25 mL/min flow rate. The received reaction mixture was evaporated *in vacuo*, then purified by flash column chromatography using hexane:ethyl acetate 10:1 to 1:1 eluent. The structure of the products was characterized by ^{1}H NMR and HR-MS.

*(E)-N-(4-hydroxy-3-methoxybenzyl)-8-methylnon-6-enamide (**6a**)*

White solid, yield: 72% (1.10 g), productivity: 3.30 g/h, m. p.: 60–64 °C, ^{1}H NMR ($CDCl_3$) δ 0.95 (d, J_{HH} = 6.7, 6H, $\underline{C}H_3CH\underline{C}H_3$), 1.38 (p, J_{HH} = 7.6, 2H, $COCH_2CH_2\underline{C}H_2$), 1.65 (p, J_{HH} = 7.6, 2H, $COCH_2\underline{C}H_2$), 1.98 (dt, J_{HH} = 7.5, J_{HH} = 6.9, 2H, $\underline{C}H_2CH{=}CH$), 2.20 (t, J_{HH} = 7.6, 2H, $CO\underline{C}H_2CH_2$), 2.16–2.25 (m, 1H, $CH_3\underline{C}HCH_3$), 3.86 (s, 3H, $O\underline{C}H_3$), 4.34 (d, J_{HH} = 5.6, 2H, $Ar\underline{C}H_2NH$), 5.27–5.40 (m, 2H, $\underline{C}H{=}\underline{C}H$), 5.87 (s, 2H, NH and OH signals overlap), 6.74 (dd, J_{HH} = 8.0, J_{HH} = 1.8, 1H, $\underline{C_6}H$), 6.79 (d, J_{HH} = 1.9, 1H, $\underline{C_2}H$), 6.85 (d, J_{HH} = 8.1, 1H, $\underline{C_5}H$); HRMS (ESI) m/z: 306.2055 $[M+H]^+$ (calcd. for $C_{18}H_{28}NO_3$, 306.2069).

*N-(4-hydroxy-3-methoxybenzyl)-8-methylnonanamide (**6b**)*

White solid, yield: 75% (1.15 g), productivity: 3.45 g/h, m. p.: 63–65 °C, ^{1}H NMR ($CDCl_3$) δ 0.78 (d, J_{HH} = 6.4, 6H, $\underline{C}H_3CH\underline{C}H_3$), 1.02–1.11 (m, 2H, $(CH_3)_2CH\underline{C}H_2$), 1.15–1.28 (m, 6H, $(CH_3)_2CHCH_2(\underline{C}H_2)_3$), 1.38–1.47 (m, 1H, $CH_3\underline{C}HCH_3$), 1.53–1.62 (m, 2H, $COCH_2\underline{C}H_2$), 2.12 (t, J_{HH} = 7.6, 2H, $CO\underline{C}H_2CH_2$), 3.80 (s, 3H, $O\underline{C}H_3$), 4.28 (d, J_{HH} = 5.6, 2H, $Ar\underline{C}H_2NH$), 5.67 (s, 2H, NH and OH signals overlap), 6.68 (d, J_{HH} = 8.1, 1H, $\underline{C_6}H$), 6.73 (s, 1H, $\underline{C_2}H$), 6.79 (d, J_{HH} = 8.1, 1H, $\underline{C_5}H$); HRMS (ESI) m/z: 308.2239 $[M+H]^+$ (calcd. for $C_{18}H_{30}NO_3$, 308.2226).

*N-(4-hydroxy-3-methoxybenzyl)nonanamide (**6c**)*

White solid, yield: 90% (1.32 g), productivity: 3.96 g/h, m. p.: 54–56 °C, ^{1}H NMR ($CDCl_3$) δ 0.87 (t, J_{HH} = 6.9, 3H, $\underline{C}H_3CH_2$), 1.21–1.33 (m, 10H, $CH_3\underline{C}H_2\underline{C}H_2\underline{C}H_2\underline{C}H_2\underline{C}H_2$), 1.64 (p, J_{HH} = 7.3, 2H, $\underline{C}H_2CH_2CO$), 2.19 (t, J_{HH} = 7.6, 2H, $\underline{C}H_2CO$), 3.87 (s, 3H, $O\underline{C}H_3$), 4.34 (d, J_{HH} = 5.6, 2H, $Ar\underline{C}H_2NH$), 5.76 (s, 2H, NH and OH signals overlap), 6.75 (dd, J_{HH} = 8.0, J_{HH} = 1.8, 1H, $\underline{C_6}H$), 6.80 (d, J_{HH} = 1.8, 1H, $\underline{C_2}H$), 6.86 (d, J_{HH} = 8.0, 1H, $\underline{C_5}H$); HRMS (ESI) m/z: 294.2081 $[M+H]^+$ (calcd. for $C_{17}H_{28}NO_3$, 294.2069).

4. ^{1}H NMR analysis for Job's plot method

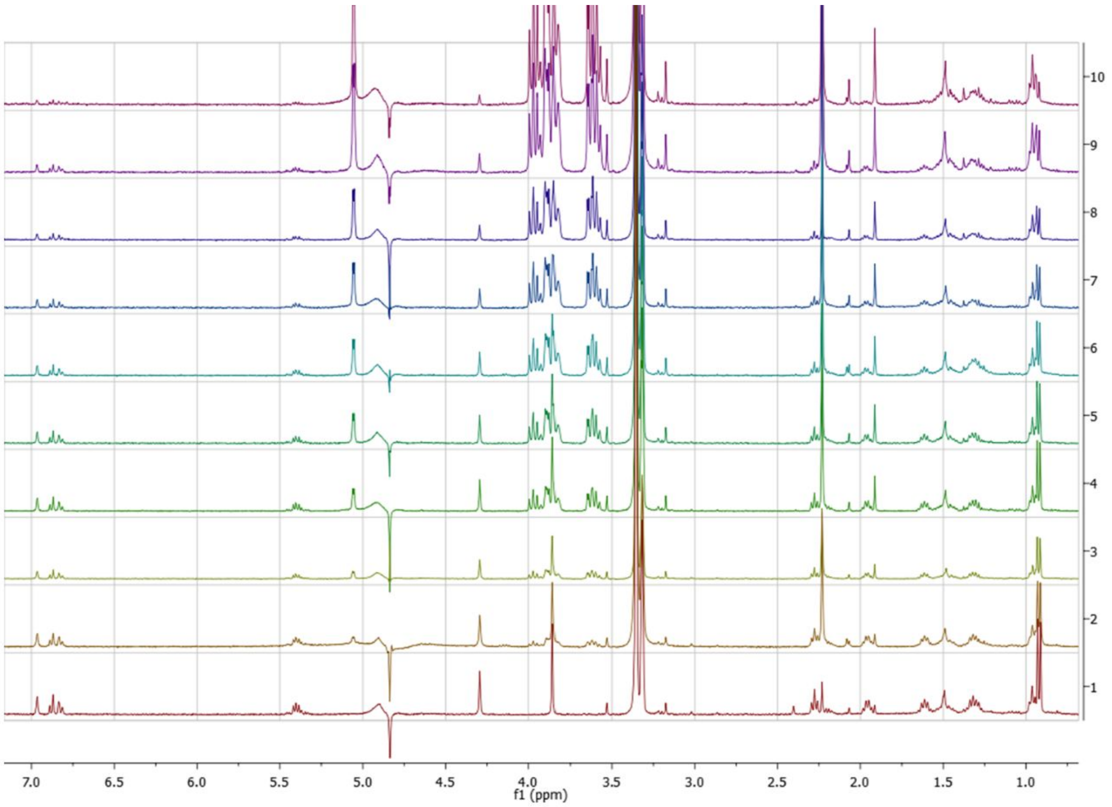

Fig. S1. Capsaicin (**6a**)-α-CD ^{1}H NMR spectra for Job's plot method (full spectra)

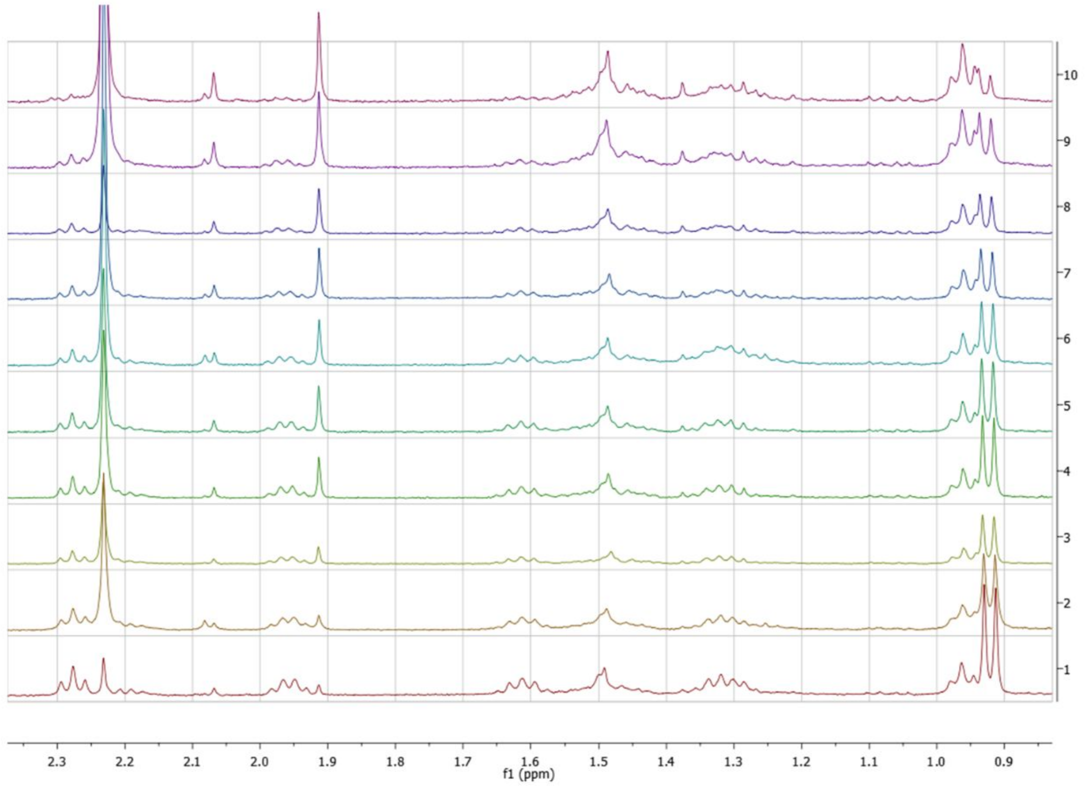

Fig. S2. Capsaicin (**6a**)-α-CD ^{1}H NMR spectra for Job's plot method (methyl region)

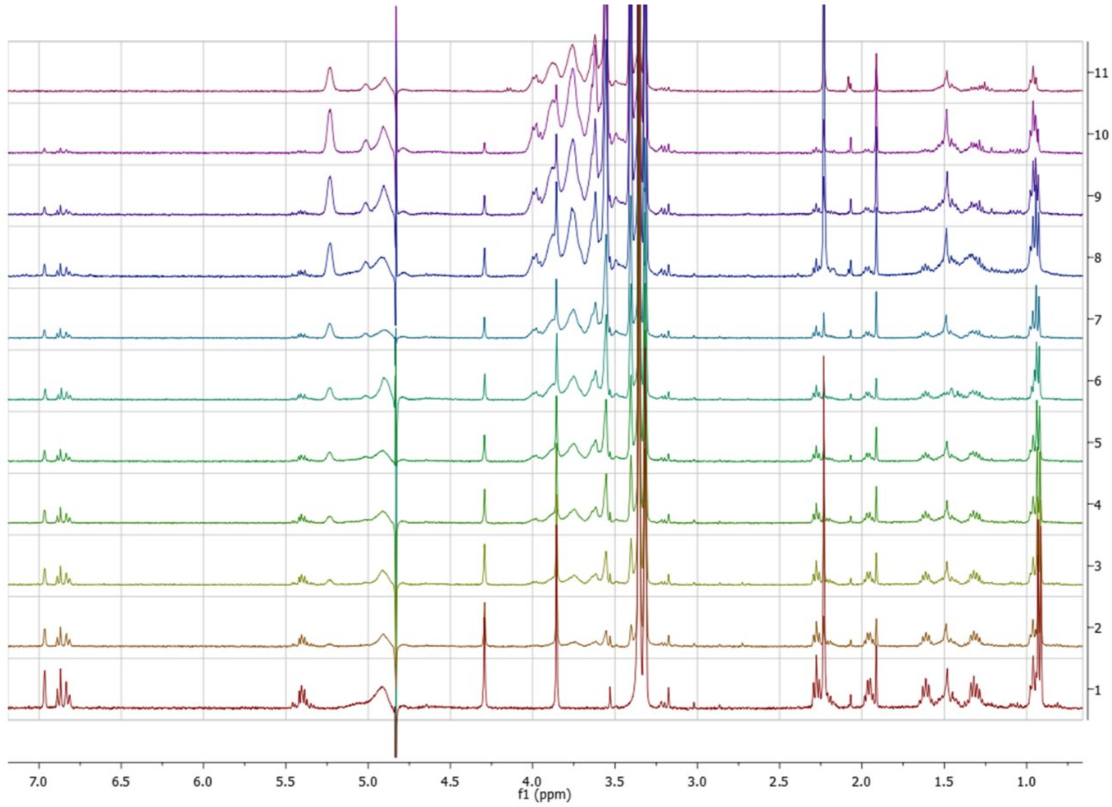

Fig. S3. Capsaicin (**6a**)-RAME-α-CD ^{1}H NMR spectra for Job's plot method (full spectra)

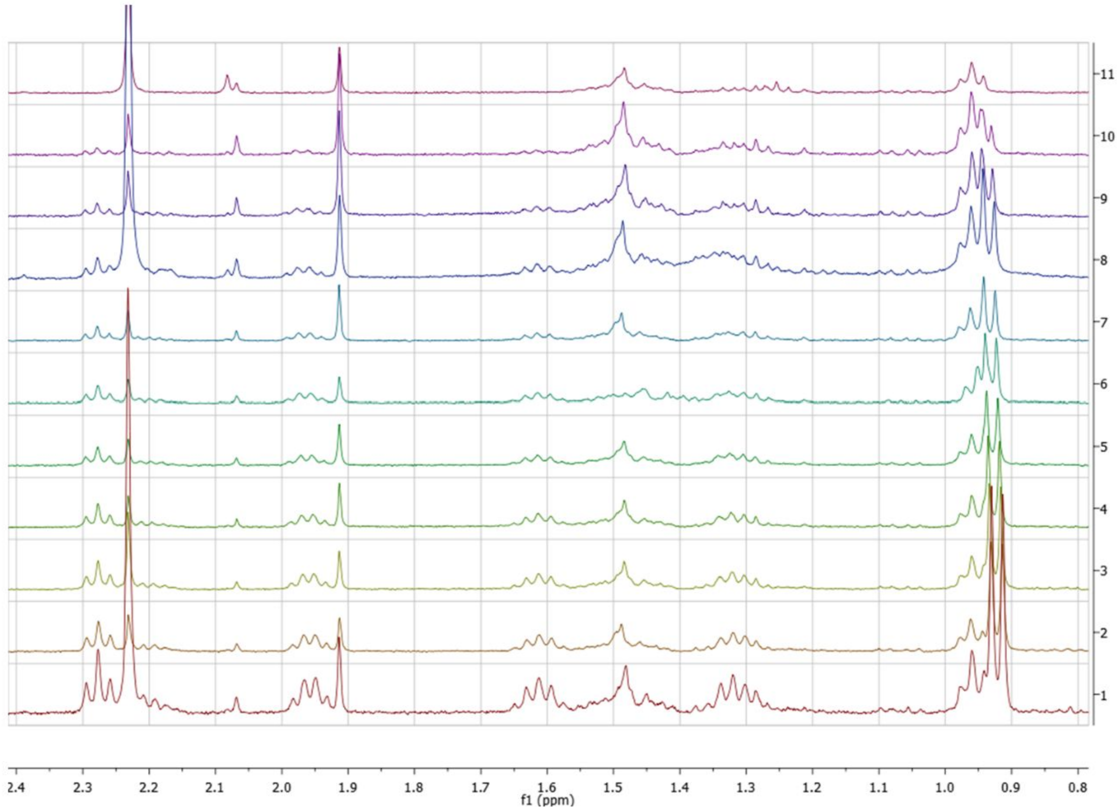

Fig. S4. Capsaicin (**6a**)-RAME-α-CD ^{1}H NMR spectra for Job's plot method (methyl region)

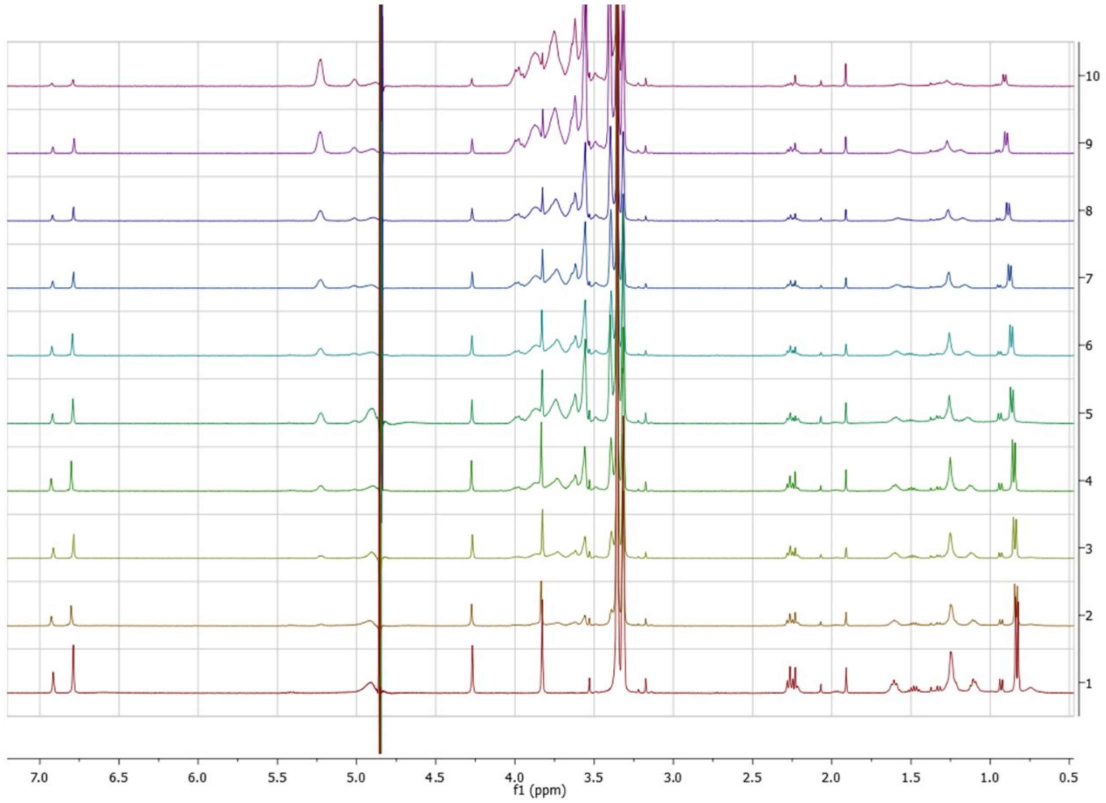

Fig. S5. Dihydrocapsaicin (**6b**)-RAME-α-CD ^{1}H NMR spectra for Job's plot method (full spectra)

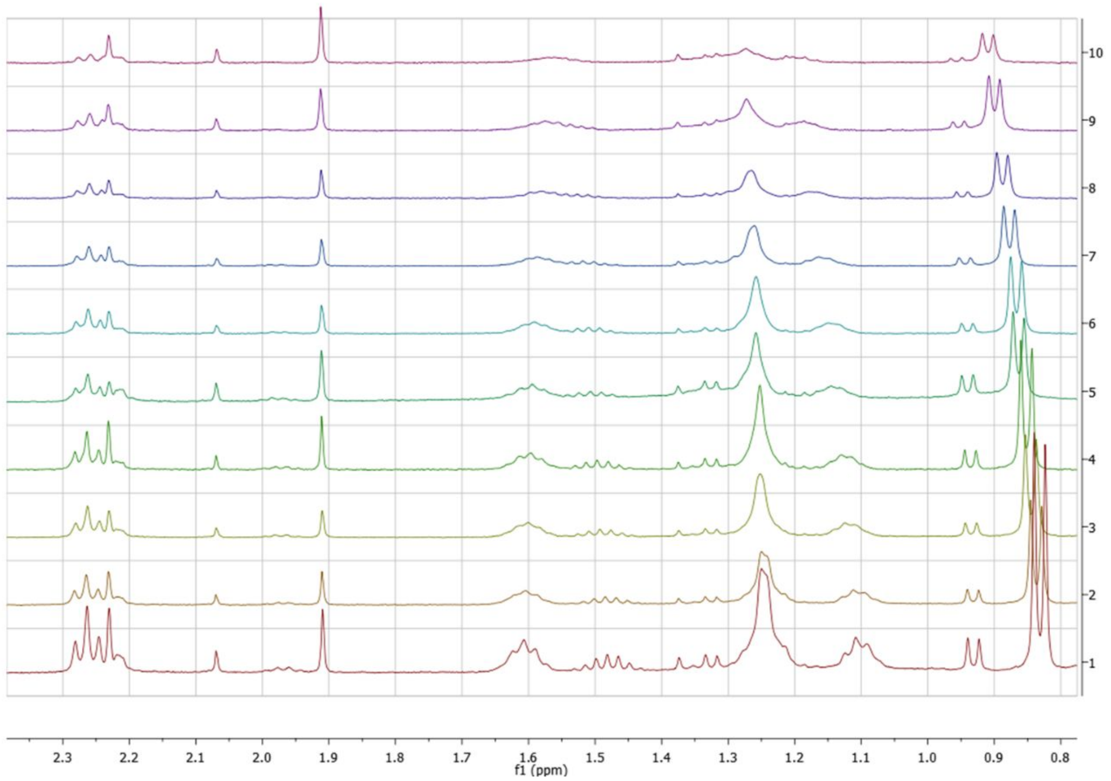

Fig. S6. Dihydrocapsaicin (**6b**)-RAME-α-CD ^{1}H NMR spectra for Job's plot method (methyl region)

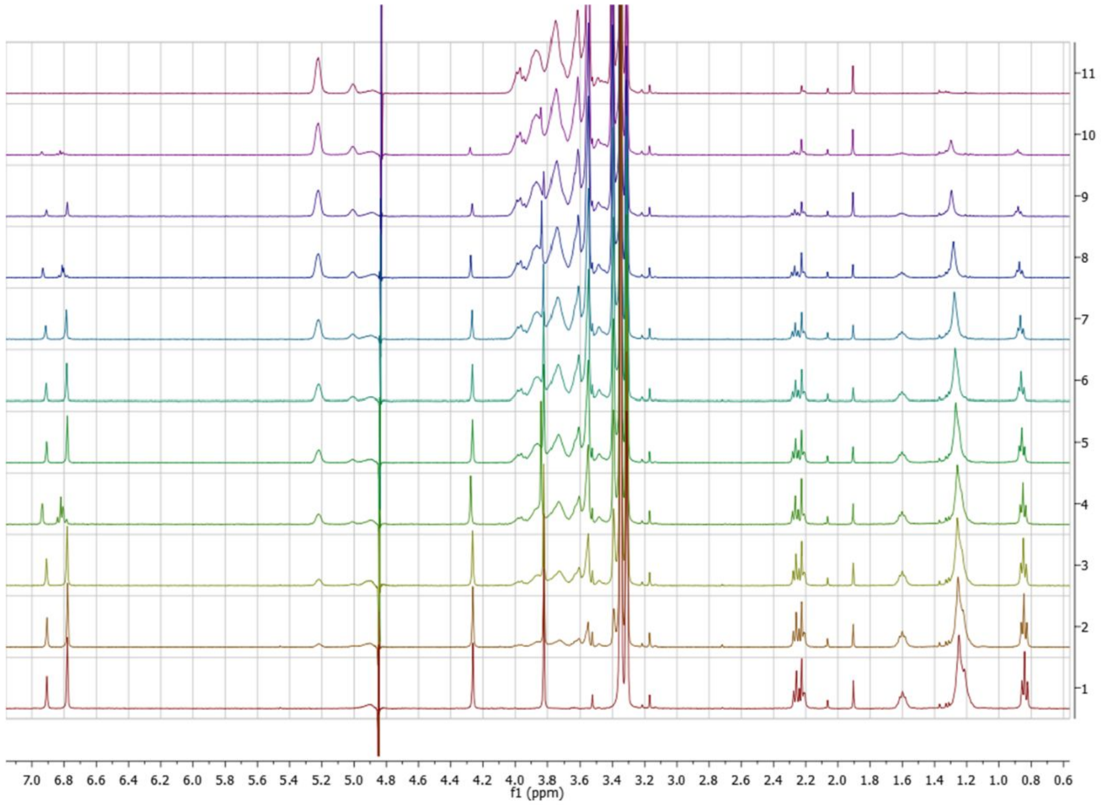

Fig. S7. Nonivamide (**6c**)-RAME-α-CD ^{1}H NMR spectra for Job's plot method (full spectra)

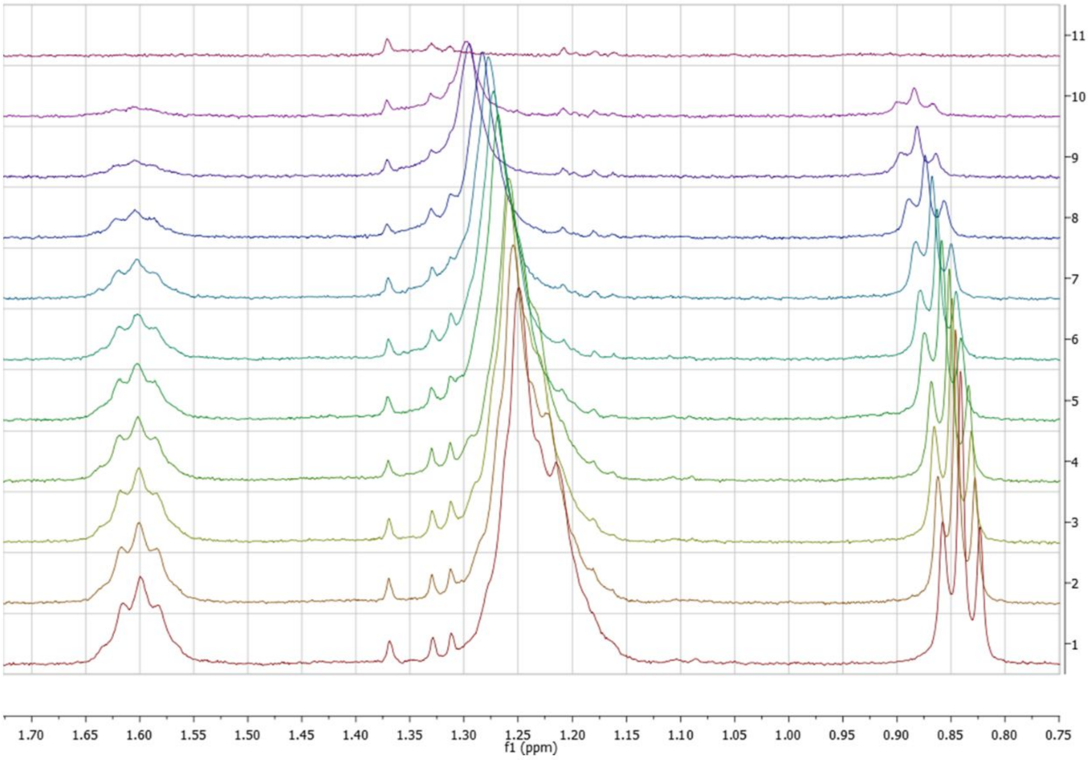

Fig. S8. Nonivamide (**6c**)-RAME-α-CD ^{1}H NMR spectra for Job's plot method (methyl region)

5. ROESY analysis

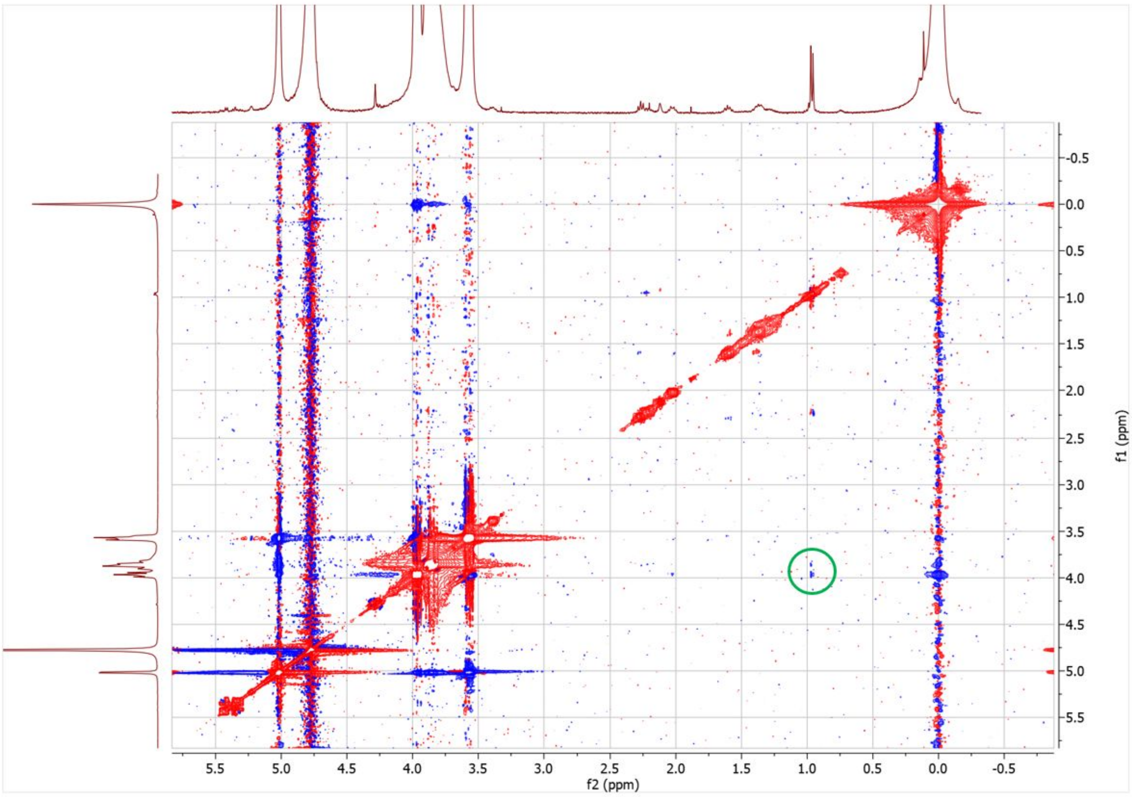

Fig. S9. The detected cross-peaks in the capsaicin (**6a**)–α-CD complex

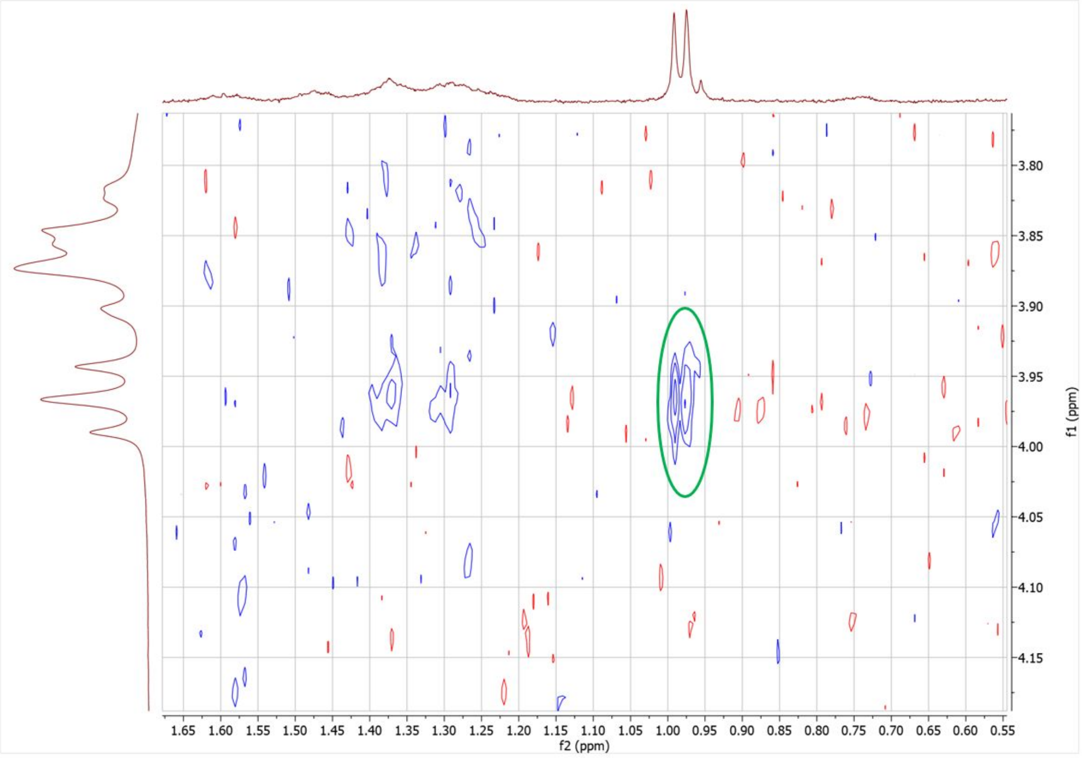

Fig. S10. The detected cross-peaks in the dihydrocapsaicin (**6b**)–α-CD complex

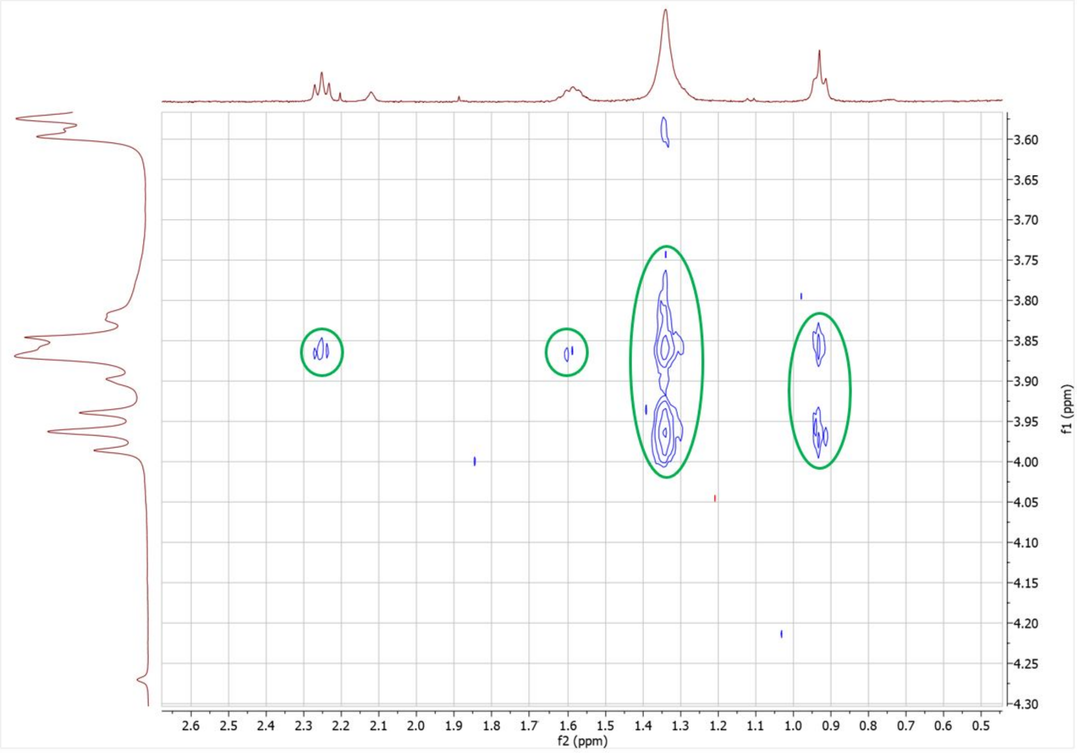

Fig. S11. The detected cross-peaks in the nonivamide (**6c**)–α-CD complex

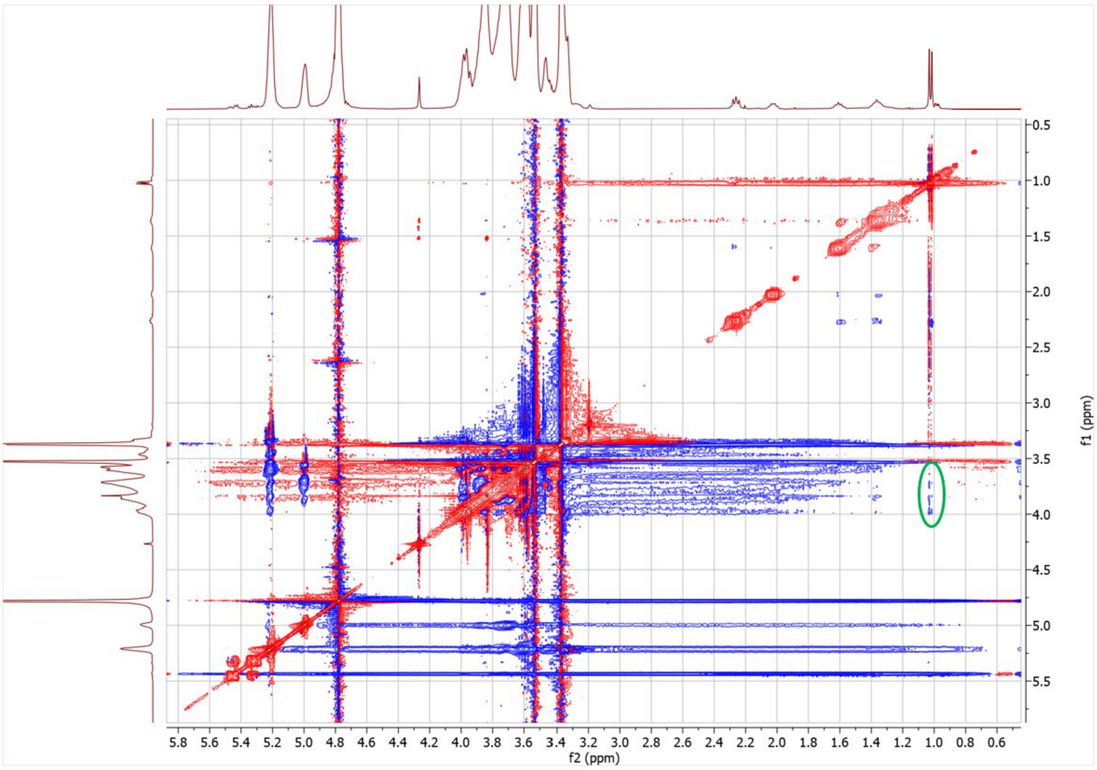

Fig. S12. The detected cross-peaks in the capsaicin (**6a**)–RAME-α-CD complex

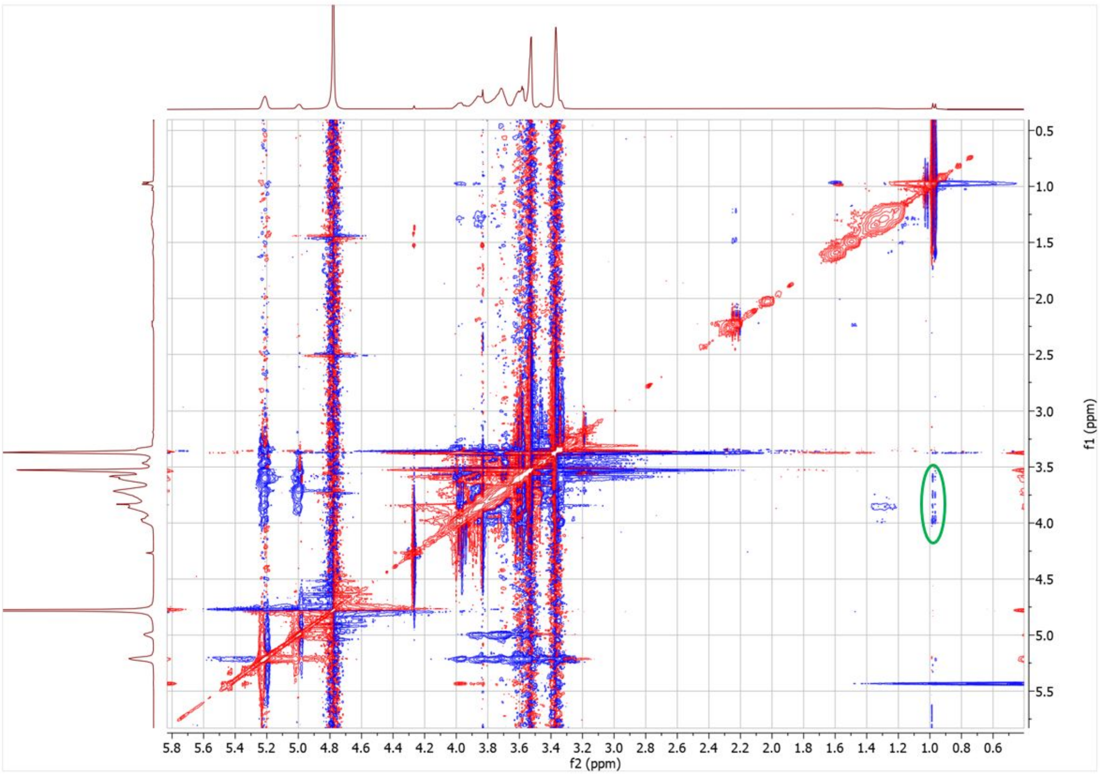

Fig. S13. The detected cross-peaks in the dihydrocapsaicin (**6b**)–RAME-α-CD complex

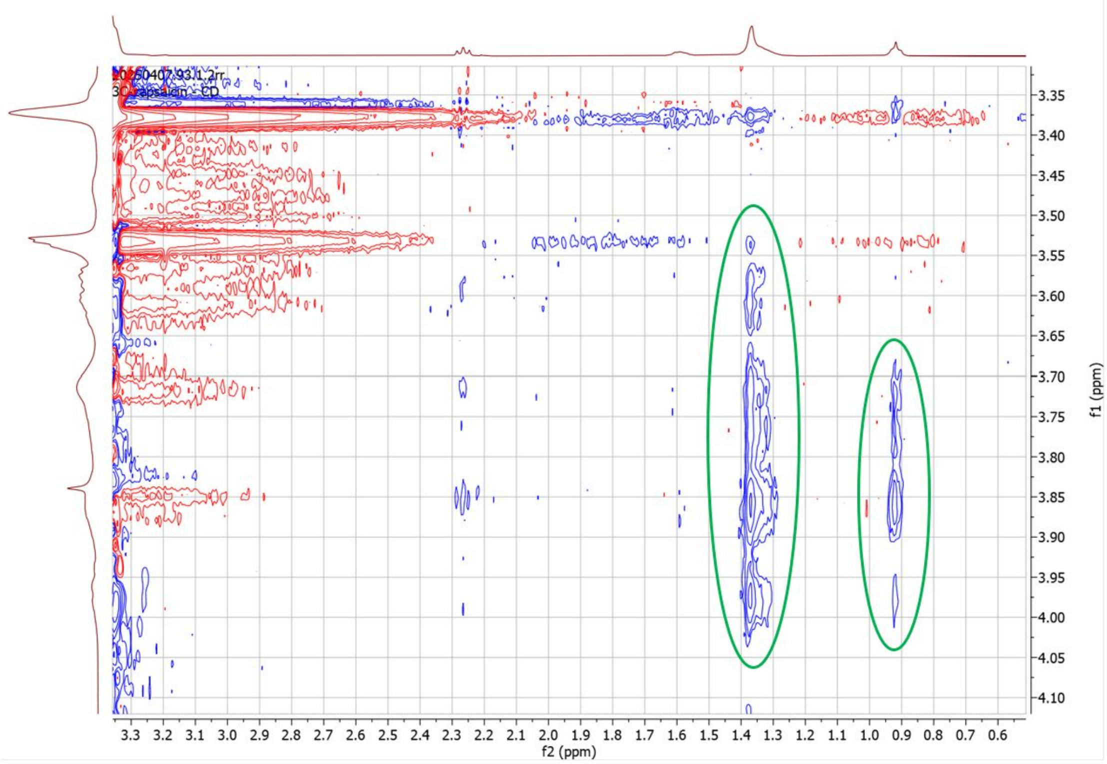

Fig. S14. The detected cross-peaks in the nonivamide (**6c**)–RAME-α-CD complex

6. References

(S1) A. Khan, F. Naaz, R. Basit, D. Das, P. Bisht, M. Shaikh, B. A. Lone, Y. R. Pokharel, Q. N. Ahmed, S. Parveen, I. Ali, S. K. Singh, G. Chashoo, S. Shafi, 1,2,3-Triazole tethered hybrid capsaicinoids as antiproliferative agents active against lung cancer cells (A549). *ACS Omega* **2022**, *7*, 32078–32100.

(S2) H. Kaga, M. Miura, K. Orito, A facile procedure for synthesis of capsaicin. *J. Org. Chem.* **1989**, *54*, 3477.

(S3) M. Xie, H. Wu, J. Bian, S. Huang, Y. Xia, Y. Qin, Z. Yan, Synthesis and biological evaluation of capsaicin analogues as antioxidant and neuroprotective agents. *RSC Adv.* **2023**, *13,* 32150.

(S4) M. J. Orosz, B. Rávai, B. Mátravölgyi, E. Bálint, Flow synthesis of capsaicin and capsaicinoid analogues, *ACS Sustain. Chem. Eng.* **2024**, *12*, 7913.
